# Off-The-Grid Variational Sparse Spike Recovery: Methods and Algorithms

**DOI:** 10.3390/jimaging7120266

**Published:** 2021-12-06

**Authors:** Bastien Laville, Laure Blanc-Féraud, Gilles Aubert

**Affiliations:** 1 Université Côte d’Azur, CNRS, Inria, I3S, Morpheme Project, 06900 Sophia Antipolis, France; 2 Université Côte d’Azur, CNRS, LJAD, 06000 Nice, France

**Keywords:** off-the-grid optimisation review, inverse problems, sparse spike localisation, super-resolution, fluorescence microscopy, SMLM, functional analysis

## Abstract

Gridless sparse spike reconstruction is a rather new research field with significant results for the super-resolution problem, where we want to retrieve fine-scale details from a noisy and filtered acquisition. To tackle this problem, we are interested in optimisation under some prior, typically the sparsity i.e., the source is composed of spikes. Following the seminal work on the generalised LASSO for measures called the *Beurling-Lasso* (BLASSO), we will give a review on the chief theoretical and numerical breakthrough of the off-the-grid inverse problem, as we illustrate its usefulness to the super-resolution problem in Single Molecule Localisation Microscopy (SMLM) through new reconstruction metrics and tests on synthetic and real SMLM data we performed for this review.

## 1. Introduction

In this paper, we propose to conduct a comprehensive review on the so-called *off-the-grid variational methods* to solve the sparse spike recovery problem. We will exhibit the main theoretical and numerical results in the literature, underlining the interest of these methods for various domains dealing with inverse problems. As part of this review and our former work on gridless methods, we developed an implementation of the more consistent numerical methods with a focus on efficiency and computation time. With this implementation, we were able to apply off-the-grid method to fluorescence microscopy super-resolution problem. The codes and the computed result are an addition to the off-the-grid literature, and constitute further evidence supporting the relevance of this domain in the inverse problem field.

Loosely speaking, inverse problems consist in the reconstruction of the causes from the consequences. The problem is generally ill-posed, meaning that existence, uniqueness, and stability of a solution(s) is (are) not guaranteed. A case arising in numerous fields such as image or signal processing, telecommunications, machine learning, super-resolution, etc. is the *sparse spike problem*. It consists of the reconstruction of spikes located on a domain X from an acquisition *y*, with the prior of sparsity on the cause; or in layman terms, the source is composed of a few spikes. This includes sources such as stars in astronomy, fractures in seismology, etc. A spike is typically modelled by a Dirac measure $a\delta_{x}$ with amplitude a∈C and position x∈X. All the difficulty lies in the estimation of the number *N* of spikes, of their amplitudes ${\left(a_{i}\right)}_{i=1}^{N}$ and their positions ${\left(x_{i}\right)}_{i=1}^{N}$. Hence, the goal is to reconstruct the measure $m=\sum_{i=1}^{N}a_{i}\delta_{x_{i}}$ only from a few number of observations *y* in a Hilbert space H (typically $L^{2}\left(X\right)$) linked to *m* through an operator Φ accounting for deterioration of the input (blur, downsizing by the sampling) such as $y\overset{\mathrm{def}.}{=}\Phi m+w$ where w∈H is an additive noise. The reconstruction of the spikes may be *off-the-grid* i.e., the positions ${\left(x_{i}\right)}_{i=1}^{N}$ are not constrained on a grid hence ${\left(x_{i}\right)}_{i=1}^{N}$ are not limited to a finite set of values: this allows interesting new mathematical insights and guarantees for the reconstruction, at the cost of some challenges for the numerical implementation. The general sparse spike problem is encountered in many situations, such as:

- compressed sensing domain [1], where one wants to recover a *s*-sparse vector $v\in C^{N}$ from *M* measurements $Av_{0}$ where $A\in C^{M\times N}$;
- machine learning, sketching mixtures, etc. For example, we desire to fit a probability distribution with respect to given data. The point is to estimate parameters $\left(a_{i}\right)\in R^{N}$ and $\left(x_{i}\right)\in X^{N}$ of a mixture $\sum_{i=1}^{N}a_{i}\varphi \left(x_{i}\right)$ of *N* elementary distributions described by φ. For instance, one wants to retrieve the means $\mu_{i}\in R$ and standard deviations $\sigma_{i}\in R^{+}$ of a Gaussian mixture, see [2] for more insights on this question:
- deep learning such as training neural networks with a single hidden layer [3];
- signal processing, for instance low rank tensor decomposition for *Direction of Arrival* estimation through sensor array (multiple sampling points);
- super-resolution, a rather central problem in image processing. Roughly speaking, it consists of the reconstruction of details from an altered input of signal/image. It includes a classic physical operator of acquisition such as Fourier measurements, Laplace transform or Gaussian convolution.

The latter item will be our case of interest in the sparse spike problem for this paper. All the difficulty stems from the degradation in the acquisition process, which entails in general two things: a deterioration by the system of acquisition, typically modelled by the *Point Spread Function* in imagery which acts as a low-pass filter sensor acquisition which results in sampling and pollution by noise of different types, characterised by densities such as Gaussian, Poisson, etc. To sum up, we want to reconstruct the correct number of spikes with correct amplitudes and positions in the continuous setting from a noisy and filtered discrete acquisition. It can be tackled from the theoretical point of view by either the variational approach or Prony’s method:

- Prony’s method and its variants such as MUSIC (MUltiple SIgnal Classification), ESPRIT (Estimation of Signal Parameters by Rotational Invariance Techniques) or Matrix Pencil, which recover the signal source from Fourier measurements in a noiseless 1D setting. It consists of the decomposition of the signal onto a basis of exponentials with different amplitudes, damping factors, frequencies, and phase angles to match the observed data. The results are compelling in the 1D noiseless case, and can be extended to a multivariate and noisy context, but still these methods lack of versatility since they cannot be sometimes extended to the context of interest. Thus, we will not consider this approach in this paper;
- variational approach which does not impose any particular structure on the acquisition operator, which can be adapted to any type of noise and does not need any prior on the number of point sources [4]. The key idea is to solve the inverse problem by finding among all possible signal sources the one minimising an objective function called *the energy*, formulated as a trade-off between a fidelity data term and a regularisation term, typically enforcing the sparsity prior here.

Then, there are two types of variational approaches: the discrete and the off-the-grid. In the discrete setting, one seeks to recover the spikes on a prescribed fine grid, typically with more points than the acquisition image. Indeed, we call coarse grid for the low-resolved acquisition, and fine grid for the finer (by a so-called super-resolution factor $q\in N^{*}$) grid of the reconstruction. Thus, it consists of a finite dimensional problem, where the positions of the spikes must lie on a grid G of *L* points meshing the domain X. This problem is a problem of sparse vectors reconstruction, and it can be tackled by enforcing sparsity through minimisation of the $\ell_{1}$ norm of the unknown vector. This is known as the LASSO [5] or the Basis-pursuit problem, defined as the variational problem with tuning parameter λ>0 controlling the trade-off between fidelity to the data and enforcement of the prior:

(LASSO) $$\begin{array}{c} \min_{a\in R^{L}}\underset{\mathrm{data}\;\mathrm{term}}{\underset{︸}{{\left\|y-\Phi_{L}a\right\|}_{H}^{2}}}+\underset{\mathrm{sparsity}\;\mathrm{prior}}{\underset{︸}{\lambda {\left\|a\right\|}_{1}}} \end{array}$$

where $\Phi_{L}:R^{L}\to H$ is the acquisition operator with a vector of size *L* as an input and H is a Hilbert space. A grid is useful to epitomise the concept of sparsity in the case of spikes: indeed, sparsity is just the fact that only a few points of the *L* grid have a non-zero value. Moreover, since a computer can only store array and vector quantities, it seems rather fair to work with finite dimensional problem, even for the theoretical analysis. However, how does one choose the discretisation? A grid with a step-size too small yields numerical instabilities [6] while choosing a step-size that is too large leads to round-off errors. Moreover, one would like to localise the spikes as precisely as possible without having to rely on a grid: a discretisation of positions would necessarily convey approximation on positions. The appropriate mathematical objects to get rid of these discretisation drawbacks is to represent a collection of spikes with Dirac measures, an element of the space of Radon measures $M\left(X\right)$. The operator of acquisition is now $\Phi :M\left(X\right)\to H$, the sparsity is enforced by a norm on $M\left(X\right)$ called the TV-norm. This variational problem is called the BLASSO (for Beurling LASSO):

(BLASSO) $$\begin{array}{c} \min_{m\in M\left(X\right)}\underset{\mathrm{data}\;\mathrm{term}}{\underset{︸}{{\left\|y-\Phi m\right\|}_{H}^{2}}}+\underset{\mathrm{sparsity}\;\mathrm{prior}}{\underset{︸}{\lambda {\left\|m\right\|}_{\mathrm{TV}}}}. \end{array}$$

**Figure 1:**
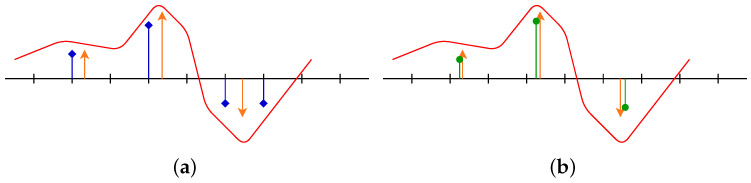
(**a**) Discrete reconstruction, which can be seen as spikes with support constrained on a grid; (**b**) off-the-grid reconstruction, the spikes can move continuously on the line. The red line is the acquisition *y*, orange spikes are the source (the cause we want to retrieve), blue spikes are discrete reconstruction constrained on a grid and green can move freely since it is off-the-grid. Note that, when a source spike is between two grid points, two spikes will be recovered in the discrete reconstruction.

In this latter setting, the spikes can move continuously on the domain X: a comparison between the discrete and the off-the-grid reconstruction is given in Figure 1. The off-the-grid setting can be seen as the limit of the discrete case with a finer and finer grid [7].

This shift from the discrete domain to the continuous setting called *off-the-grid* or *gridless* leads to some crucial mathematical insights, in particular a sharp signal-dependent criterion for stable spikes recovery [8], the *minimum* separation distance (see the next section). Obviously, some difficulties arise also due to the infinite dimension and the lack of algebraic properties of the set of optimisation. The comparison between discrete and gridless settings may be summed up by:

- the discrete problem is tackled by LASSO, through the minimisation of a convex function defined on a fine grid i.e., a convenient finite dimension Hilbert $R^{L}$ space. Due to the $\ell_{1}$ norm, there are some cases where the sparsity is not properly enforced: one can then replace the $\ell_{1}$ norm by the non-continuous pseudo-norm $\ell_{0}$, but this yields an NP-hard combinatory non-convex problem. There exists some continuous relaxation of $\ell_{0}$ such as CEL0 [9], but, due to the non-convex aspect, the problem is still hard from the theoretical and numerical point of view. Despite the lack of guarantees, there are numerous algorithms to compute the solution of LASSO or its $\ell_{0}$ relaxed variant;
- the off-the-grid problem is treated by BLASSO, a convex functional defined on $M\left(X\right)$. The convex property is handy from the theoretical point of views as it leads to some crucial insights on the existence/uniqueness/support estimation w.r.t. noise, at the cost of the set of optimisation, namely $M\left(X\right)$ a Banach (no Hilbertian structure so no straightforward proximal algorithm) infinite dimensional and non-reflexive space for the strong topology (convergence results are then essentially on the weak-* topology). Despite this lack of algebraic properties, one has currently a wide range of algorithms to tackle this problem, such as root-finding or greedy algorithms.

Gridless reconstruction can then be evaluated through suitable metrics, namely the Flat Metric based on optimal transport of measures. This metric assesses the quality of the reconstruction and can be applied straightforwardly to off-the-grid and even discrete reconstruction outputs.

In the following, we give a review on the key results in the variational off-the-grid domain. The paper is organised in three sections, namely:

- the variational analysis of the space $M\left(X\right)$, the properties and the guarantees of reconstruction concerning the sparse spike problem are now quite well-documented [8,10,11,12] and will be recalled in the theoretical Section 2;
- multiple strategies were considered to numerically tackle BLASSO, the more compelling will be presented and put into context in the numerical Section 3;
- interesting practical applications and new metrics have been considered for the gridless method, such as the SMLM super-resolution; these results are shown and discussed in Section 4.

At the end of each paragraph, a grey box (beginning either with ‘summary’ or ‘shorthand’) like this one will recall the main results highlighted in the section. Please refer to it for a quick summary.

## 2. A Theoretical Background for Gridless Spike Recovery

In the following, X denotes the ambient space where the positions of the spikes live. We suppose X is a subset of $R^{d}$ such that its interior $\overset{˚}{X}$ is a submanifold of dimension $d\in N^{*}$ [13]. This setting encompasses $X=R^{d}$, the torus $X=T^{d}\overset{\mathrm{def}.}{=}R^{d}/Z^{d}$, any compact with non-empty interior, etc. The reader is invited to take a look at the Table A1 to remind the notations.

### 2.1. What Is a Measure?

As we have stated in the section above, the Dirac measure is the proper object to describe a spike not constrained on a finite set of positions. This object is not a function, since one cannot exhibit any integrable equivalence class satisfying the properties of the Dirac (see below). Thus, one should considerate the notion of Radon measure, a formal extension of functions. From a distributional standpoint, it is a subset of the distribution space $D^{\prime }\left(X\right)$, namely the space of linear forms over the space of test functions D(X) i.e., smooth functions (continuous derivatives of all orders) compactly supported. This functional approach consists in the definition of a measure as a linear form on some function space, namely:

**Definition** **1** **(Evanescent continuous function on** X **).** *We call $C_{0}\left(X,Y\right)$ the set of continuous functions with zero at infinity (or evanescent), namely all the continuous map ψ:X→Y such that:*

$$\begin{array}{c} \forall \varepsilon >0,\exists K\subset X\;\mathrm{compact},\;\underset{x\in X\backslash K}{\sup}{\psi (x)}_{Y}\leq \varepsilon . \end{array}$$

When Y=R, we will simply write $C_{0}\left(X\right)$. Since we dispose of a suitable test functions space, we need to precise the notion of duality at stake in this review.

**Definition** **2** **(Topological dual space).** *If E is a topological vector space, we denote $E^{*}$ its topological dual i.e., the space of all continuous linear forms ψ:E→R. The pairing between an element ϕ∈E and a map $\psi \in E^{*}$ is denoted by the bilinear mapping ${\langle \phi ,\psi \rangle }_{E\times E^{*}}\overset{\mathrm{def}.}{=}\psi \left(\phi \right)$ called the duality bracket.*

This notion allows us to define the Radon measure through duality in the following definition.

**Definition** **3** **(Set of Radon Measures).** *We denote $M\left(X\right)$ the set of real signed Radon measures on X of finite masses. It is the topological dual of $C_{0}\left(X\right)$ with supremum norm ${\;\|\cdot \|\;}_{\infty ,X}$ by the Riesz–Markov representation theorem. It can also be defined as the topological dual of the space of continuous function $C\left(X\right)$ if X is compact [14]. Thus, a Radon measure m is a continuous linear form evaluated on functions $f\in C_{0}\left(X\right)$, with for $m\in M\left(X\right)$ the duality bracket denoted by ${\langle f,m\rangle }_{C_{0}\left(X\right)\times M\left(X\right)}=\int_{X}f\;dm$.*

The term ‘signed’ refers to the generalisation of the concept of (positive) measure, by allowing the quantity ${\langle f,m\rangle }_{C_{0}\left(X\right)\times M\left(X\right)}$ to be negative. We can define in the same way the space of real non-negative Radon measures $M^{+}\left(X\right)$ dual of $C_{0}\left(X,R^{+}\right)$ and the space of complex Radon measures $M_{C}\left(X\right)$ dual of $C_{0}\left(X,C\right)$. Classic examples of Radon measures are:

- the Lebesgue measure of dimension d∈N;
- the Dirac measure $\delta_{z}$ centred in z∈X, also called the δ-peak. For all $f\in C_{0}\left(X\right)$, one has ${\langle f,\delta_{z}\rangle }_{C_{0}\left(X\right)\times M\left(X\right)}=f\left(z\right)$;
- discrete measures $m_{a,x}\overset{\mathrm{def}.}{=}\sum_{i=1}^{N}a_{i}\delta_{x_{i}}$ where N∈N, *a*$\in C^{N}$, *x*$\in X^{N}$.

Since $C_{0}\left(X\right)$ is a Banach space, $M\left(X\right)$ is complete [11] by endowing it with its dual norm called the total variation (TV) norm, defined for $m\in M\left(X\right)$ by:

$$\begin{array}{c} |m|\left(X\right)\overset{\mathrm{def}.}{=}\sup\left(\int_{X}f\;dm,f\in C_{0}\left(X\right),{\left\|f\right\|}_{\infty ,X}\leq 1\right). \end{array}$$

The TV norm of a measure is also called its *mass*. One can note that, in the case of a *discrete measure* defined as before $m_{a,x}\overset{\mathrm{def}.}{=}\sum_{i=1}^{N}a_{i}\delta_{x_{i}}$, one has $|m_{a,x}|\left(X\right)={\left\|a\right\|}_{1}$.

The interested reader might take a look at the Appendix B.1 for more details on some functional analysis notions and results.

**Summary:** we model a spike by a Dirac measure, an element of the Radon measure spaces $M\left(X\right)$. This space is defined by duality, it is endowed by the TV-norm and is complete. It is, however, infinite dimensional and non-reflexive (see Appendix B.1), this poses additional difficulties to be taken into account in the optimisation.

### 2.2. Observations

Let us introduce the space where the acquired data live. We will denote by H this Hilbert space; for the instance of images, $H=L^{2}\left(X\right)$. Let $m\in M\left(X\right)$ be the source measure, we call *acquisition* y∈H the result of the *forward/acquisition map* $\Phi :M\left(X\right)\to H$ evaluated on *m*, with measurement kernel φ:X→H:

(1) $$\begin{array}{c} y\overset{\mathrm{def}.}{=}\Phi m=\int_{X}\varphi \left(x\right)\;dm\left(x\right). \end{array}$$

The latter integral ought to not be confused with the duality bracket ${\langle f,m\rangle }_{C_{0}\left(X\right)\times M\left(X\right)}$$=\int_{X}f\left(x\right)\;dm\left(x\right)$ mentioned in Definition 3 above. Indeed, while f(x)∈R for x∈X, we have φ(x)∈H: the integral in (1) is then a *Böchner integral* [15] i.e., the proper notion to deal with vector valued map. It is valid as long as φ is continuous and bounded [3,13].

**Remark** **1.** *Measures are objects that generalise functions at the cost of losing some of their properties. Thus, one cannot define a product of measures (what would be the square of the Dirac?) and one ought to be aware of some caveats concerning the functions of measure: these functionals need to be at most (sub)linear in order to be well-defined [16].*

In the following, we will impose $\varphi \in C^{2}\left(X,H\right)$. Let us also define the adjoint operator of $\Phi :M\left(X\right)\to H$ in the weak-* topology, namely the map $\Phi^{*}:H\to C_{0}\left(X\right)$. It is defined for all x∈X and p∈H by $\Phi^{*}\left(p\right)\left(x\right)={\langle p,\varphi (x)\rangle }_{H}$. The choice of φ and H depends on the physical process of acquisition; indeed, generic measurement kernels are:

- convolution kernel with typically $H=L^{2}\left(X\right)$ and $\forall x\in X,\varphi \left(x\right)\overset{\mathrm{def}.}{=}\left(s\mapsto \tilde{\varphi }\left(s-x\right)\right)\in H$, for the PSF $\tilde{\varphi }\in C_{0}^{2}\left(R^{d}\right)$. One has, for instance, the Gaussian kernel, centred in c∈X with spread σ>0, defined by $s\mapsto \tilde{\varphi }\left(s-c\right)\overset{\mathrm{def}.}{=}1/\sqrt[{d/2}]{2\pi \sigma^{2}}\;e^{-{\left\|s-c\right\|}_{2}^{2}/2\sigma^{2}}$;
- Fourier kernel with cut-off frequency $f_{c}\in N$ and $H=C^{2f_{c}+1}$, for x∈X=T in 1D:
  $$\begin{array}{c} \varphi \left(x\right)={\left(e^{2i\pi kx}\right)}_{\left|k\right|\leq f_{c}}; \end{array}$$
- Laplace kernel [4] for non-negative weighting function $\xi \in C\left(X\right)$ specific to the physical acquisition process and $H=L^{2}\left(R^{+}\right)$: $\forall x\in X,\;\varphi \left(x\right)\overset{\mathrm{def}.}{=}\left(s\mapsto \xi \left(x\right)e^{-sx}\right)\in H$.

These three kernels correspond to various physical context of imagery, hence they are encountered in multiple acquisition process, such as Nuclear Magnetic Resonance spectroscopy (Fourier), SMLM super-resolution (convolution), MA-TIRF (Laplace), etc.

We will now on use the following notation for the discrete forward map: let $x=(x_{1},\cdots ,x_{N})$ and $a\in R^{N}$: $\Phi_{x}\left(a\right)\overset{\mathrm{def}.}{=}\sum_{i=1}^{N}a_{i}\varphi \left(x_{i}\right).$

**Shorthand:** an acquisition living in the Hilbert space H of a measure *m* is the quantity Φm. Φ is the forward operator, completely defined by a kernel φ specific to the physical context of imagery.

### 2.3. An Off-The-Grid Functional: The BLASSO

Let $m_{a_{0},x_{0}}\overset{\mathrm{def}.}{=}\sum_{i=1}^{N}a_{0,i}\delta_{x_{0,i}}$ be the source measure with amplitudes $a_{0}$$\in R^{N}$ and positions $x_{0}$$\in X^{N}$, the sparse spike problem is to recover this measure from the acquisition $y\overset{\mathrm{def}.}{=}\Phi m_{a_{0},x_{0}}+w$, where w∈H is an additive noise, typically white Gaussian noise. To tackle this problem, we use the following convex functional [11,17] also called the BLASSO, which stands for Beurling-LASSO:

(𝒫_λ_(*y*)) $$\begin{array}{c} \underset{m\in M\left(X\right)}{\mathrm{argmin}}T_{\lambda }\left(m\right)\overset{\mathrm{def}.}{=}\frac{1}{2}{\left\|y-\Phi (m)\right\|}_{H}^{2}+\lambda \left|m\right|\left(X\right) \end{array}$$

with regularisation parameter λ>0 which accounts for the trade-off between fidelity and sparsity of the reconstruction. The name BLASSO was coined in the work of [17,18] according to the link between the *Generalised Minimal Extrapolation* (GME) problem where one seeks to reconstruct a Radon measure from several observations on its Fourier coefficients, and the work [19] of the Norwegian mathematician Beurling, which coincides with GME in the case of a Fourier forward operator.

The BLASSO in a noiseless setting writes down:

(𝒫_0_(*y*_0_)) $$\begin{array}{c} \underset{\Phi m=y_{0}}{\mathrm{argmin}}\left|m\right|\left(X\right)\;\mathrm{with}\;y_{0}=\Phi m_{a_{0},x_{0}}. \end{array}$$

BLASSO is genuinely linked with its discrete counterpart the (LASSO) [8]: one can formally see BLASSO as the functional limit of LASSO on a finer and finer grid. If the LASSO problem exhibits existence and uniqueness of the solution, what can one say for its off-the-grid counterpart? First of all, let us observe that:

- m↦|m|(X) is lower semi-continuous w.r.t. the weak-* convergence (see Appendix B.1 for more insights);
- Φ is continuous from the weak-* topology of $M\left(X\right)$ to the weak topology of H.

Thus, one can establish the existence of solutions to (𝒫_λ_(*y*)) thanks to convex analysis results, as proved in [11].

**Summary:** the sparse spike problem is tractable thanks to the convex functional on $M\left(X\right)$ called the BLASSO and denoted by (𝒫_λ_(*y*)). With $m\in M\left(X\right)$ as an input, it consists of a data term comparing observed data versus Φm, and a regularisation accounting for sparsity prior through the TV-norm of *m*. Existence of solutions of the BLASSO is known and proved.

The difficulties now lie in the following questions:

(1) what are the conditions to recover a sparse measure, within a certain noise regime? Is the *minimum* unique?
(2) under which conditions can we retrieve exactly the number of spikes, the amplitude, and the positions; when do we have support stability?
(3) how can we tackle numerically the infinite dimensional and non-reflexive nature of the space $M\left(X\right)$?

In order to address these points, we need to introduce some notions of convex analysis in the following subsection.

### 2.4. Dual Problems and Certificates

The BLASSO in Equation (𝒫_λ_(*y*)) above is a minimisation problem with a convex functional. Then, we can apply Ekeland–Temam ([20] Remark 4.2) results and define a dual problem which writes down for p∈H (see Appendix B.2 for the proof):

(𝒟_λ_(*y*)) $$\begin{array}{c} \underset{{\left\|\phi^{*}p\right\|}_{\infty ,X}\leq 1}{\mathrm{argmax}}{\langle y,p\rangle }_{H}-\frac{\lambda }{2}{\left\|p\right\|}_{H}^{2} \end{array}$$

which can be recast as the projection onto a closed convex [11,18]:

(𝒟_λ_’(*y*)) $$\begin{array}{c} \underset{{\left\|\phi^{*}p\right\|}_{\infty ,X}\leq 1}{\mathrm{argmax}}{\left\|\frac{y}{\lambda }-p\right\|}_{H}^{2} \end{array}$$

Fenchel’s duality between (𝒫_λ_(*y*)) and (𝒟_λ_(*y*)) is proved in [11]. Therefore, any solution $m_{\lambda }$ of (𝒫_λ_(*y*)) is linked [8] to the unique solution $p_{\lambda }$ of (𝒟_λ_(*y*)) by the extremality conditions:

(2) $$\begin{array}{c} \left\{\begin{array}{c} \Phi^{*}p_{\lambda }\in \partial |m_{\lambda }|\left(X\right),\\ -p_{\lambda }=\frac{1}{\lambda }\left(\Phi m_{\lambda }-y\right) \end{array}\right. \end{array}$$

where ∂|·|(X) is the sub-differential of the TV norm. Indeed, since the total variation is not differentiable (as the $\ell^{1}$ norm) but lower semi-continuous w.r.t. the weak-*topology, we use its sub-differential which for $m\in M\left(X\right)$ identifies to:

(3) $$\begin{array}{c} \partial |m|\left(X\right)=\left\{\eta \in C_{0}\left(X\right);{\left\|\eta \right\|}_{\infty ,X}\leq 1\;\mathrm{and}\;\int_{X}\eta \;dm=\left|m\right|\left(X\right)\right\}. \end{array}$$

Elements of this subgradient are called *certificate*. Thanks to strong duality, one can define peculiar certificates called the *dual certificates* [10].

**Definition** **4.** *We call $\eta_{\lambda }\overset{\mathrm{def}.}{=}\Phi^{*}p_{\lambda }$, where $p_{\lambda }$ satisfies (2), a dual certificate of $m_{\lambda }$.*

It is a certificate since $\Phi^{*}p_{\lambda }\in \partial |m_{\lambda }|\left(X\right)$, and it is called *dual* because it verifies the second extremality (2) condition: it is thus defined by the dual solution $p_{\lambda }$. Loosely speaking, a dual certificate $\eta_{\lambda }$ is associated with a measure $m_{\lambda }$, and it *certifies* that the measure $m_{\lambda }$ is a *minimum* of the BLASSO. For instance, if there exist solutions of (𝒫_λ_(*y*)) of the form $m_{\lambda }\overset{\mathrm{def}.}{=}\sum_{i=1}^{N}a_{i}\delta_{x_{i}}$, the support satisfies [8] for all 0≤i≤N : $|\eta_{\lambda }|\left(x_{i}\right)=1$.

In the same fashion, one has the link between a solution $m_{0}$ of the noiseless BLASSO (𝒫_0_(*y*_0_)) and its certificates $\eta_{0}$, which are not unique in general. Then, in the rest of the document, we will refer to $\eta_{0}$ as the minimal norm certificate i.e., the dual certificate $\eta_{0}$ with minimal *supremum* norm ${\left\|\eta_{0}\right\|}_{\infty ,X}$. It is shown in [8] that this minimal norm certificate $\eta_{0}$ has important properties, since it somehow drives the stability of the recovered spike locations when the additive noise is small, in particular how close they are to the positions of the true measure $m_{a_{0},x_{0}}$: see Definition 6 in the section below.

**Summary:** we defined the primal problem in the former section, thanks to convexity, we can define the dual problem of the BLASSO. A solution $m_{\lambda }$ of the BLASSO and a solution $p_{\lambda }$ of the dual problem are linked through extremality condition. The dual solution $p_{\lambda }$ defines the dual certificate, an element of the subgradient specified by $\eta_{\lambda }=\Phi^{*}p_{\lambda }$: the dual certificate $\eta_{\lambda }$ *certifies* that $m_{\lambda }$ is a solution of the BLASSO. We can then establish more precise conditions on the uniqueness/support recovery.

### 2.5. Support Recovery Guarantees

We will address in this section the first two questions we have laid down, namely existence, uniqueness, and support recovery conditions. A classical tool to establish some recovery properties lies in the notion of the *minimum* separation distance.

**Definition** **5** **(Minimum separation distance).** *The minimum separation distance is a characterisation of the support of the discrete measure $m_{a_{0},x_{0}}$ by:*

$$\begin{array}{c} \Delta \left(m_{a_{0},x_{0}}\right)\overset{\mathrm{def}.}{=}\min_{i\neq j}\left|x_{0,i}-x_{0,j}\right|. \end{array}$$

The reconstruction condition is driven by this minimum separation distance, itself determined by the type of measure (complex, real, real non-negative) and the type of forward operator.

- if the operator is an acquisition of the Fourier spectrum within $\left[-f_{c},f_{c}\right]$ with frequency cut-off $f_{c}$ for $X=T^{d}$ the *d*-torus in the noiseless setting, it is necessary that $\Delta \left(m_{a_{0},x_{0}}\right)≳\frac{2}{f_{c}}$ if the source measure is complex [10]. Upon a few conditions [21], one can weaken it to $\Delta \left(m_{a_{0},x_{0}}\right)≳\frac{1.26}{f_{c}}$, and $\Delta \left(m_{a_{0},x_{0}}\right)≳\frac{1.87}{f_{c}}$ if the source measure is real [10];
- regardless of the operator Φ [17,22], there is no condition on the separation for a real **positive** source measure in the noiseless setting; however, stability constant explodes when $\Delta (m_{a_{0},x_{0}})\to 0$.

These results are important but do not provide a sharp characterisation of the recovery in the presence of noise; however, we expect to find noise in the images we deal with and therefore to be limited by this noise regime. To account for this effect, we need to add some conditions on the ground-truth measure; following the work of [8], we introduce:

**Definition** **6** **(Non-degenerate source condition).** *The source $m_{a_{0},x_{0}}$ verifies the NDSC (Non-Degenerate Source Condition) if:*

- *there exists η∈ImΦ∗ such that $\eta \in \partial |m_{a_{0},x_{0}}|\left(X\right)$;*
- *$\forall s\in X\backslash \cup_{i=1}^{N}\left\{x_{0,i}\right\},\left|\eta_{0}\left(s\right)\right|<1$;*
- *∀i∈⟦1,N⟧, the Hessian matrix $\nabla^{2}\eta_{0}\left(x_{0,i}\right)\in R^{d\times d}$ is invertible.*

The first condition amounts to assuming that $m_{a_{0},x_{0}}$ is a solution to (𝒫_0_(*y*_0_)) and there exists a solution to its dual problem. If the two latter conditions are matched, we say that $\eta_{0}$ is *not degenerate*. This allows us to write the main result of [8], namely:

**Theorem** **1** **(Noise robustness [8]).** *Let $\Gamma_{x_{0}}$ the N×N matrix defined by $\Gamma_{x_{0}}\overset{\mathrm{def}.}{=}{\left(\varphi \left(\;\cdot \;-x_{0,i}\right),\varphi^{\prime }\left(\;\cdot \;-x_{0,i}\right)\right)}_{i=1}^{N}$. Assume that $\Gamma_{x_{0}}$ has full column rank and that $m_{a_{0},x_{0}}$ verifies the NDSC. Then, there exists $\alpha >0,\lambda_{0}>0$ such that for all $0\leq \lambda \leq \lambda_{0}$ and w such that ‖w‖≤αλ; there exists N pairings $\left(a_{\lambda ,i},x_{\lambda ,i}\right)$ such that $m_{\lambda }\overset{\mathrm{def}.}{=}\sum_{i=1}^{N}a_{\lambda ,i}\delta_{x_{\lambda ,i}}$ is the unique solution of Ref. (𝒫_λ_(*y*)) composed of exactly N spikes. In particular, for $\lambda =1/\alpha {\left\|w\right\|}_{H}$, we have the control over the discrepancies:*

$$\begin{array}{c} \forall i\in ⟦1,N⟧:\;\left\|x_{\lambda ,i}-x_{0,i}\right\|=𝒪\left({\left\|w\right\|}_{H}\right)\;\;\mathrm{and}\;\;\left|a_{\lambda ,i}-a_{0,i}\right|=𝒪\left({\left\|w\right\|}_{H}\right). \end{array}$$

Under the Non-Degenerate Source Condition, for λ and ${\left\|w\right\|}_{H}^{2}/\lambda$ small enough, one can reconstruct a measure with the same number of spikes as the ground-truth measure $m_{a_{0},x_{0}}$. Furthermore, the reconstructed measure (weak-*)converges to the ground-truth measure when the noise level drops to 0. The authors of [8] also introduce the notion of vanishing derivatives precertificate. The $\eta_{0}$ certificate is indeed hard to compute from the dual problem of (𝒫_0_(*y*_0_)). Because of the constraint ${{\left\|\eta \right\|}_{0}}_{\infty ,X}\leq 1$, the precertificate allows for leveraging this computation by solving instead a linear system. The interested reader is advised to take a glance at this article among other ones [8,22] for these new concepts.

**Shorthand:** the minimum separation distance criterion is used to assess recovery possibilities in the noiseless setting. In a low regime of noise, a theorem states that the source measure $m_{a,x}$ composed of *N* spikes can be recovered through BLASSO, with a control over the discrepancies (amplitudes/positions) between the reconstructed and the source measures.

We were therefore able to establish some guarantees on the reconstruction of the source measures in the presence of noise. In the next section, we propose to address the third question and to discuss strategies to compute the numerical solution of the inverse problem; a difficult task requiring accounting for the difficulties of the optimisation space.

## 3. Numerical Strategies to Tackle the BLASSO

The BLASSO problem 𝒫_λ_(*y*) is an optimisation over the set of Radon measures, an infinite dimensional and non-reflexive space. We recall that it writes down:

(𝒫_λ_(*y*)) $$\begin{array}{c} \underset{m\in M\left(X\right)}{\mathrm{argmin}}T_{\lambda }\left(m\right)\overset{\mathrm{def}.}{=}\frac{1}{2}{\left\|y-\Phi (m)\right\|}_{H}^{2}+\lambda \left|m\right|\left(X\right). \end{array}$$

A naive approach would be to enforce the measure *m* to be supported on a fine grid ${\left(p_{i}\right)}_{i}^{L}$ which is equivalent to solving the LASSO problem:

$$\begin{array}{c} \min_{a\in R^{L}}{\left\|y-\Phi_{L}a\right\|}_{H}^{2}+{\left\|a\right\|}_{1} \end{array}$$

with the discrete operator $\Phi_{L}a\overset{\mathrm{def}.}{=}\sum_{i=1}^{L}a_{i}\varphi \left(p_{i}\right)$ and φ the kernel of the forward operator. This approach conveys numerous cons: for instance, the solution of the LASSO, in small noise regime and when the step size tends to 0, contains pairs of spikes around the true one [6,7]. Furthermore, refining the step size leads to a worse conditioning of the forward operator, accounting for numerical difficulties. The following classes of algorithms better account for the infinite dimensional nature of $M\left(X\right)$. We present in detail the three methods with the most established results in the literature [13,18,23]. Before describing these methods, let us remark that there also exist some promising avenues, such as the projected gradient descent [24,25]. It relies on an over parametrised initialisation i.e., a discrete measure with numerous δ-peaks compared to the ground-truth, then one applies a gradient descent on the amplitudes and positions of the over parametrised measure combined at each step with a projection on a set of positions constraints to enforce the separation of the spikes. This projection can be replaced by a ‘heuristic’ which boils down to the merging of δ-peaks that are not enough separated [25].

### 3.1. Semi-Definite Recasting and Hierarchy

Semi-definite programming was one of the first schemes solving the BLASSO in the specific case of a Fourier acquisition on the 1D torus $T^{1}$ [10,12,17,18]. Before explaining in layman terms the SDP scheme, let us first introduce and detail the relevant quantities for this section. Let d=1 be the dimension of the interior of X, let us study the case where the forward operator denoted by $F_{n}$ (and not Φ for this section) is a Fourier coefficient measurement up to some cut-off frequency $f_{c}\in N$, with $n=2f_{c}+1$ the number of measurements. We have $F_{n}:M_{C}\left(X\right)\to C^{n}$ and, for a discrete measure $m_{a,x}\overset{\mathrm{def}.}{=}\sum_{j=1}^{N}a_{j}\delta_{x_{j}}$, it writes down $F_{n}\left(m_{a,x}\right)={\left(\sum_{j}a_{j}e^{2i\pi kx_{j}}\right)}_{\left|k\right|\leq f_{c}}$ and its adjoint operator $F_{n}^{*}:C^{n}\to C_{0}\left(X,C\right)$ is for s∈X:

(4) $$\begin{array}{c} \forall c\in C^{n},\;F_{n}^{*}\left(c\right)\left(s\right)={\langle c,{\left(e^{2i\pi ks}\right)}_{\;\left|\;k\;\right|\;\leq f_{c}}\rangle }_{C^{n}}=\sum_{\left|k\right|\leq f_{c}}c_{f_{c}+k}\;e^{2i\pi ks}. \end{array}$$

This method is based on semi-definite programming (SDP) for efficiently computing the *minima* of BLASSO. It stems from the Hilbert approach [26] when one globally decomposes the objective function into simple pieces, atoms. The solution of the dual problem of (𝒫_λ_(*y*)), denoted here $\left({𝒟}_{\lambda }^{ℱ}(y)\right)$, is a polynomial *p* linked to a certificate by $F_{n}^{*}p$: the idea then is the reconstruction of the dual certificate as a linear sum of trigonometric polynomials [18], which is enough to find the measure associated with this reconstructed certificate. This associated measure is a solution to the BLASSO. The dual problem, on the other hand, is tractable thanks to a semi-definite programming approach.

Since $F_{n}^{*}p$ is a trigonometric polynomial for any $p\in C^{n}$ by the definition above, one can recast the constraint ${\left\|F_{n}^{*}p\right\|}_{\infty ,X}\leq 1$ (imposed by definition of a certificate, see Equation (3)) and rewrite it as the intersection of the cone of positive semi-definite matrices {A:A⪰0} with an affine hyperplane [10,27]. Hence, the Fenchel dual problem of (𝒫_λ_(*y*)) for the Fourier forward operator $F_{n}$:

(𝒟λℱ(y))maxp∈nRe{y,p}−λ2‖p‖ℋ2constrainedby‖Fn∗p‖∞,X≤1

with Hermitian product $\left\{\;\cdot \;,\;\cdot \;\right\}$, has the equivalent formulation [27]

maxp∈Cn,Q∈Cn×nRe{y,p}−λ2‖p‖H2constrainedby(𝒟∼λℱ(y))Qpp∗1⪰0and∑k=1n−jQk,k+j=δ0,jforj∈⟦1,n−1⟧

with *Q* being a Hermitian matrix and *p* a vector of coefficients (accounting for the dual variable *p*), and $\delta_{0,j}$ the Kronecker delta equal to 1 if j=0 and 0, otherwise. The choice of regulariser λ is crucial: if chosen to be too high, it will yield a solution with fewer spikes, if chosen too low, it will recover a solution with spurious spikes. This finite dimensional formulation can now be tackled with classic semi-definite programming solvers, as did the authors of [10] who proposed an algorithm of *Interior Point Method*, given in Algorithm 1. The first step reaches a solution *p*, allowing the definition of the certificate $p_{2n-2}\left(e^{2i\pi t}\right)\overset{\mathrm{def}.}{=}1-{\left|F_{n}^{*}p\right|}^{2}\left(t\right)$, where $F_{n}^{*}$ is defined in Equation (4).
**Algorithm 1.** Interior Point Method applied to the BLASSO._1_Solve
maxp∈Cn,Q∈Cn×nRey,p−λ2‖p‖H2
 subject to $\left(\begin{array}{cc} Q & p\\ p^{*} & 1 \end{array}\right)⪰0$ and $\sum_{i=1}^{n-j}Q_{i,i+j}=\delta_{0,j}$ for j=1,⋯,n−1._2_Reconstruct the support $\hat{X}$ of *m* by locating the roots of $p_{2n-2}$ on the unit circle (e.g., by computing the eigenvalues of its companion matrix)._3_Solve $\sum_{t\in \hat{X}}a_{t}e^{-2i\pi kt}=y_{k}$ to recover the amplitudes *a*.

**Figure 2:**
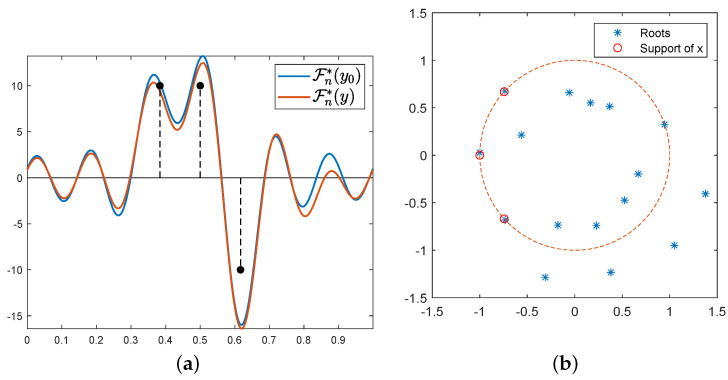
(**a**) Certificates associated with acquisition *y* and noiseless $y_{0}$, result of three δ-peaks (in black, plotted with 10 times their ground-truth amplitudes) through a Fourier measurement of cut-off frequency $f_{c}=6$; (**b**) localisation of the roots of the certificate associated with the dual *maximum*. All the roots (the three ground-truths and the spurious spike on the right) on the unit circle are interpreted as the support of the δ-peaks.

One can note the link between the dual and the primal problem, i.e., that *p*, the solution of $\left({\tilde{𝒟}}_{\lambda }^{ℱ}(y)\right)$, entails the location of the spikes: as $F_{n}^{*}p$ yields its extremal points on the support of *m* since it is the certificate of a discrete measure, note that $p_{2n-2}\left(e^{2i\pi t}\right)=1-{\left|F_{n}^{*}p\right|}^{2}\left(t\right)$ has all its roots on the unit circle, and these roots are the support of the target measure [10]. Thus, the strategy is to solve the dual problem and then to use a root-finding algorithm on the certificate $F_{n}^{*}p$ associated with the dual solution, hence reconstructing the support of the measure then the measure (after a last amplitude recover step). We present an example of the reconstruction of three Dirac measures on the 1D torus $T^{1}$ through the observed noisy data *y* and the roots of the polynomial $p_{2n-2}\left(e^{2i\pi t}\right)$ in Figure 2.

**Figure 3:**
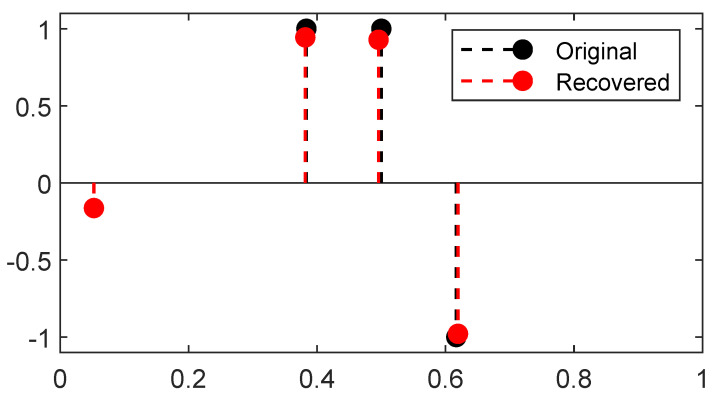
Reconstruction with the Interior Point Method for λ=1. The algorithm detected a spurious spike near 0.05; otherwise, amplitudes and positions of the peaks are correctly estimated.

This strategy is only suitable for d=1. For the multi-variate case, one needs to make use of a so-called Lasserre Hierarchy [28]. Consider the semi-definite relaxation of order *m* with $m>n=2f_{c}+1$:

maxp∈Cn,Q∈Cn×nRey,pconstrainedby0⪯Qp˜p˜∗1 where p˜k=ckifk∈[−fc,fc]d0otherwiseTr(ΘkQ)=δ0,kwithk∈⟦−m,m⟧

with $\Theta_{k}=\theta_{k_{d}}⊗\cdots ⊗\theta_{k_{1}}$, where $\theta_{k_{j}}$ and the entries of m×m elementary Toeplitz matrix are 1 on its $k_{j}$-th diagonal and 0 elsewhere, and ⊗ the Kronecker product. In a nutshell, Lasserre’s hierarchies give a sequence of nested outer SDP approximations of the cone of moments of non-negative measure. This method has been successfully applied to super-resolution in [12]. Some reconstructions in the 1D setting with a Fourier kernel are given in Figure 3, and the interested reader may find a more in-depth tutorial in the Numerical Tours on ‘Sparse spikes measures’ joined with the code used to compute the following figure (https://nbviewer.jupyter.org/github/gpeyre/numerical-tours/blob/master/matlab/sparsity_8_sparsespikes_measures.ipynb, consulted on 30 November 2021).

These methods are proved to be asymptotically exact [12]. Nonetheless, it is not known if the algorithm has finite convergence in general: one does not know when to stop the hierarchy to obtain a solution of the BLASSO [3]. This stems from the fact that non-negative trigonometric polynomials in dimension d>1 are not necessarily sums of square. Moreover, these SDP based approaches are rather limited to a certain class of measurement map Φ, typically the Fourier forward operator or at least filters with compact Fourier supports. With the two following classes of algorithms, one can better exploit the continuous setting and get rid of the discretisation drawback.

**Summary (1st algorithm):** the scheme boils down to the resolution of the dual problem, the reconstruction of the measure’s support thanks to the certificate associated with the dual solution, and finally the solving of a linear problem to yield the corresponding estimated amplitudes. This strategy can be extended to a multivariate context, but, still, it is quite restrictive on the forward operator and it does not have finite convergence in general.

### 3.2. Greedy Algorithm: The Conditional Gradient

The conditional gradient method, also called the Frank–Wolfe (FW) algorithm [29,30], aims at solving $\min_{m\in C}f\left(m\right)$ for *C* a weakly compact convex set of a topological vector space and *f* a convex and differentiable function (the differential is then denoted by d*f*). It relies on the iterative minimisation of a linearised version of *f*. Hence, the interest of this algorithm lies in the fact that it uses only the directional derivatives of *f* and that it does not require any Hilbertian structure, contrary to a classic proximal algorithm formulated in terms of Euclidean distance. We recall the definition of the conditional gradient in the pseudocode Algorithm 2 for the general problem of minimising *f*:
**Algorithm 2.** Frank–Wolfe.
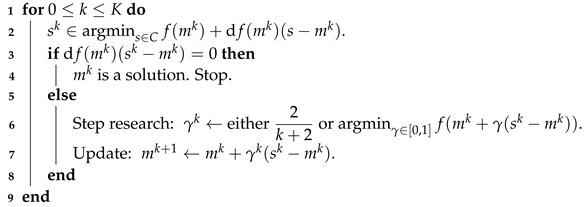


One can make the following remarks:

- the compactness assumption on *C* ensures that the argmin in step 2 is non-empty;
- in line 7, we can replace $m^{k+1}$ by any element $\hat{m}\in C$ such that $f\left(\hat{m}\right)\leq f\left(m^{k+1}\right)$ without changing the convergence properties of the algorithm;

There are, however, two problems that prevent us from applying straightforwardly this algorithm to BLASSO: $T_{\lambda }$ is not differentiable, and the optimisation set $M\left(X\right)$ is not bounded. It is thus necessary to perform an *epigraphical lift* [13,31] to reach a differentiable functional that shares the same *minimum* measures as $T_{\lambda }$:

(𝒫∼λ(y))min(r,m)∈CT˜λ(m,r)=def.12‖y−Φ(m)‖H2+λr

with the bounded set $C=\left\{\left(r,m\right)\in R^{+}\times M\left(X\right);\left|m\right|\left(X\right)\leq r\leq M\right\}$ and $M\overset{\mathrm{def}.}{=}\frac{{\left\|y\right\|}^{2}}{2\lambda }$. Even though *C* is not weakly compact, it is compact for the weak-* topology and the hypotheses for Algorithm 2 are still matched. The Frank–Wolfe algorithm is then well-defined for the energy ${\tilde{T}}_{\lambda }$, differentiable in the Fréchet sense on the Banach $R\times M\left(X\right)$. Its differential writes down:

$$\begin{array}{c} \;d{\tilde{T}}_{\lambda }:\left(r^{\prime },m^{\prime }\right)\mapsto \int_{X}\Phi^{*}\left(\Phi m-y\right)\;dm^{\prime }+\lambda r^{\prime }. \end{array}$$

Finally, one has that $m^{*}$ is a *minimum* of $T_{\lambda }$ iff $\left(|m^{*}|\left(X\right),m^{*}\right)$ minimises $\left({\tilde{𝒫}}_{\lambda }(y)\right)$, and $T_{\lambda }\left(m^{*}\right)={\tilde{T}}_{\lambda }\left(|m^{*}|\left(X\right),m^{*}\right)$. In the rest of the document, we will omit the *r*-part, and we will refer to the quantity $\left(|m^{*}|\left(X\right),m^{*}\right)$ by only $m^{*}$.

We note before that the update $m^{k+1}$ in line 7 can be replaced by any value $\hat{m}$ improving the objective function; this remark is rather interesting as it can drastically improve the convergence property of the algorithm [11,32]. Hence, an interesting improvement to the Frank–Wolfe algorithm relies in the change of the final update step by a non-convex optimisation on both the amplitudes and the positions of the reconstructed δ-peaks in a simultaneous fashion. This modification is presented in Algorithm 3.
**Algorithm 3.***Sliding Frank-Wolfe*.
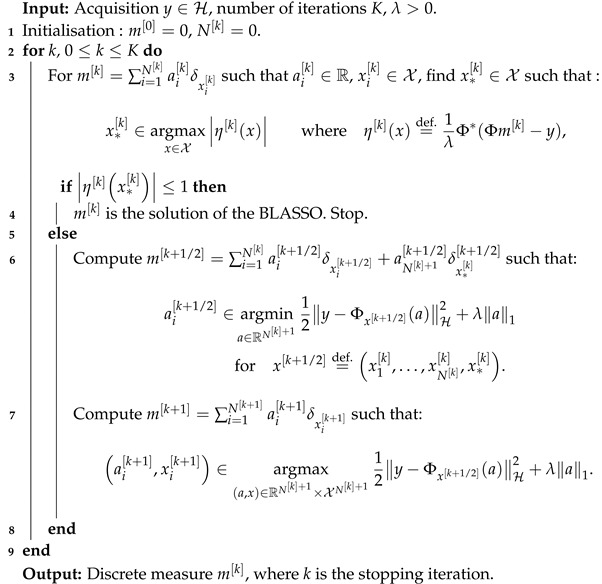


This tweak yields a theoretical convergence to the unique solution of BLASSO in a finite number of iterations, empirically a *N*-step convergence. This version is called the *Sliding Frank–Wolfe* algorithm [13], as the spike positions are sliding on the continuous domain X. The authors also proved in the same paper that the generated measure sequence $m^{\left[k\right]}$ converges towards the *minimum* for the weak-* topology.

**Figure 4:**
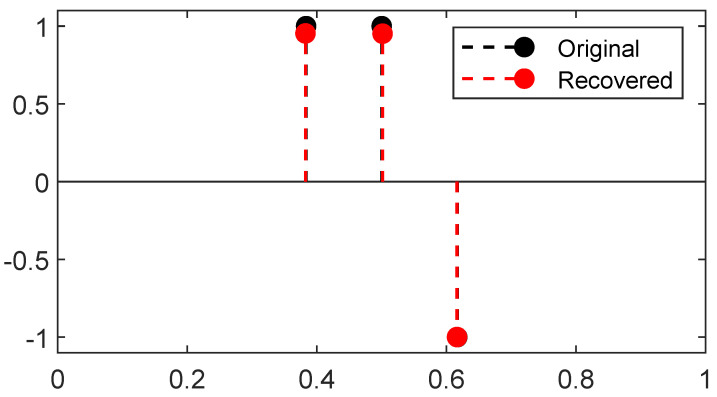
Reconstruction by *Sliding Frank-Wolfe* for a 1D Fourier operator, with the same settings (*y*, noise realisations, λ=1) as the former section. All ground-truth spikes are reconstructed, no spurious spike is detected.

A reconstruction by *Sliding Frank-Wolfe* for the same Fourier operator, ground-truth spikes, and acquisition as the latter section is plotted in Figure 4. Contrary to SDP in Figure 3, no spurious spike is reconstructed. As in the SDP method, the choice of regulariser λ is crucial: if chosen too high, it will yield a solution with fewer spikes than needed; if set too low, it will recover a solution with spurious spikes. We set λ=1 for the 1D Fourier example as in the former SDP section.

**Figure 5:**
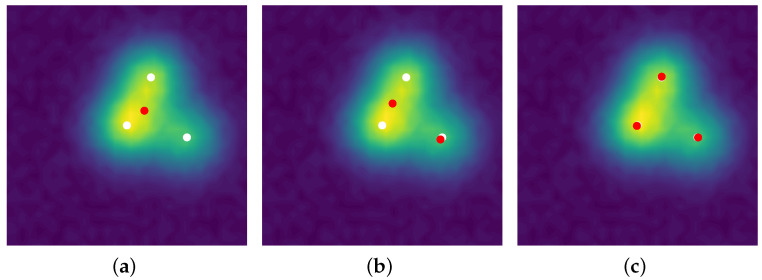
(**a**) First iterate k=0; (**b**) mid-computation k=1; (**c**) end of the computation k=2, results for SFW reconstruction on the domain $X={\left[0,1\right]}^{2}$ for the Gaussian kernel with spread-factor σ=0.1 and additive Gaussian noise of variance 0.1. All δ-peaks are successfully recovered only thanks to the acquisition, $\lambda =3\times 10^{-2}$.

The line 3 in Algorithm 3 is typically solved by a grid search, the convex step in line 5 can use a FISTA solver [33], and the non-convex step in line 6 can be tackled by a modified Broyden–Fletcher–Goldfarb–Shann method (L-BFGS-B) implementation [34]. Reconstructions in the 2D setting with a convolution kernel, similar to the SMLM conditions, are presented in Figure 5. Since luminosity is always a non-negative quantity, one can restrict [4] the SFW to build a positive measure of the cone $M^{+}\left(X\right)$, by changing:

- the stopping condition to $\eta^{\left[k\right]}\left(x_{*}^{\left[k\right]}\right)\leq 1$;
- the LASSO step is solved for $a\in R_{+}^{N^{[k]+1}}$;
- the non-convex step is solved on $R_{+}^{N^{[k]+1}}\times X^{N^{[k]+1}}$.

Hence, this modified algorithm offers a good trade-off between precision and theoretical guarantees. However, it suffers from the high computation load for one iteration, making it slow to compute. The next section algorithm is a promising alternative with easier/cheaper iteration while still taking advantage of the continuous setting.

**Shorthand (2nd algorithm):** conditional gradient method is a greedy algorithm consisting in the iterative minimisation of a linearised version of the objective convex function. This algorithm can be applied to any forward operator without restriction on the space X. Up to a modification (SFW), the Frank–Wolfe algorithm reaches a finite convergence, empirically a *N*-step convergence for a source measure with *N* spikes. The iterations, however, are computationally costly, yielding long computation time.

### 3.3. Optimal Transport Based Algorithm: The Particle Gradient Descent

All the following results are proven for a domain X with no boundaries, e.g., the *d*-dimensional torus $T^{d}$. The case described in the former sections—X is any compact of $R^{d}$—is included in this new setting, since any compact X can be periodised to yield a domain with no boundaries. The forward operator kernel φ:X→H should also be differentiable in the Fréchet sense. The least squares term in BLASSO is denoted by the more general data term $R:H\to R^{+}$, the functional $T_{\lambda }$ of the BLASSO will now be restricted to $M^{+}\left(X\right)$ and denoted *J*; its Fréchet differential at point $\nu \in M^{+}\left(X\right)$ is denoted $J_{\nu }^{\prime }$:

(5) $$\begin{array}{cc} J(\nu ) & ={\left\|y-\Phi \nu \right\|}_{H}^{2}+\lambda \left|\nu \right|\left(X\right), \end{array}$$

(6) $$\begin{array}{cc} J_{\nu }^{\prime }\left(x\right) & ={\langle \varphi \nabla (x),R\rangle }_{H}+\lambda \;\mathrm{for}\;\mathrm{all}\;x\in X \end{array}$$

**Figure 6:**
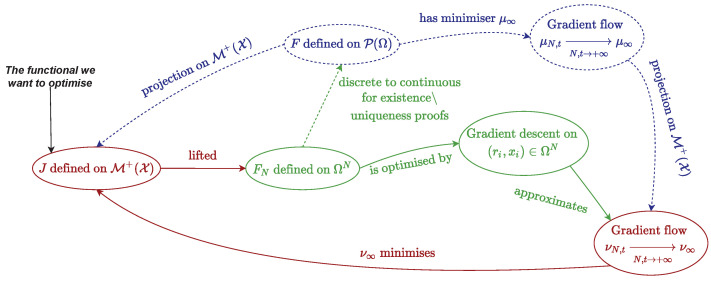
Digest of the important quantities mentioned in [3,23]: red refers to $M^{+}\left(X\right)$ quantities, green to $\Omega^{N}\overset{\mathrm{def}.}{=}\left(R^{+}\times X\right)$ and blue to the Wasserstein space $P_{2}\left(\Omega \right)$ and theoretical results. Dashed lines correspond to the theoretical section, and continuous lines indicate the numerical part.

A comprehensive guide on its computation is given in Appendix B.4. In the following, we describe the setting for non-negative measures of $M^{+}\left(X\right)$, but it can be extended in a straightforward fashion [23] to signed measures of $M\left(X\right)$ by performing the method on the positive then negative part of the signed measure (see Jordan decomposition in Appendix B.1). Figure 6 sums up the chief quantities and relations introduced in this section, the reader is advised to refer to it whenever he or she needs a global view on the optimal transport problem.

Sparse optimisation on measures through optimal transport [3,23] relies on the approximation of the ground-truth positive measure $m_{a_{0},x_{0}}$ by a ‘system of $N\in N^{*}$ particles’, i.e., an element of the space $\Omega^{N}\overset{\mathrm{def}.}{=}{\left(R^{+}\times X\right)}^{N}$. The point is then to estimate the ground-truth measure by a gradient-based optimisation on the objective function:

(7) $$\begin{array}{c} F_{N}\left(\left(r_{1},x_{1}\right),\cdots ,\left(r_{N},x_{N}\right)\right)\overset{\mathrm{def}.}{=}\|y-\frac{1}{N}\sum_{i=1}^{N}{r_{i}}^{2}\varphi \left(x_{i}\right){\|}_{H}^{2}+\frac{\lambda }{N}{r_{i}}^{2} \end{array}$$

where $\left(r_{i},x_{i}\right)$ belongs to the lifted space $\Omega \overset{\mathrm{def}.}{=}R^{+}\times X$ endowed with a metric. Hence, the hope is that the gradient descent on $F_{N}$ converges to the amplitudes and the positions of the ground-truth measure, despite the non-convexity of functional (7). The author of [23] proposes the definition of a suitable metric for the gradient of $F_{N}$, which enables separation of the variables in the gradient descent update. Let α,β be two parameters such that α>0 and β>0 and for any (r,θ)∈Ω, we define the Riemannian inner product of Ω called the *cone metric* endowing Ω as defined by $\forall \left(\delta r_{1},\delta r_{2}\right)\in R_{+}^{2}$, $\forall \left(\delta \theta_{1},\delta \theta_{2}\right)\in X^{2}$:

$$\begin{array}{c} {\langle \left(\delta r_{1},\delta \theta_{1}\right),\left(\delta r_{2},\delta \theta_{2}\right)\rangle }_{\left(r,\theta \right)}\overset{\mathrm{def}.}{=}\frac{\delta r_{1}\delta r_{2}}{\alpha }+r^{2}\frac{{\langle \delta \theta_{1},\delta \theta_{2}\rangle }_{\theta }}{\beta }. \end{array}$$

We denote by ${\langle \;\cdot \;,\;\cdot \;\rangle }_{\theta }$ the metric on the manifold X at the point θ. For the gradient of the functional $F_{N}$ for all i∈⟦1,N⟧ w.r.t.; the cone metric writes down [2,23]:

(8) $$\begin{array}{c} \left\{\begin{array}{c} \nabla_{r_{i}}F_{N}=2\alpha r_{i}\;J_{\nu }^{\prime }\left(x_{i}\right)=-2\alpha r_{i}\lambda \left(\eta_{\lambda }-1\right)\\ \nabla_{x_{i}}F_{N}=\beta \lambda \nabla J_{\nu }^{\prime }\left(x_{i}\right)=-\beta \lambda \nabla \eta_{\lambda } \end{array}\right.\;\mathrm{for}\;\nu \overset{\mathrm{def}.}{=}\sum_{i=1}^{N}{r_{i}}^{2}\delta_{x_{i}},\;\eta_{\lambda }\overset{\mathrm{def}.}{=}-J_{\nu }^{\prime }/\lambda . \end{array}$$

See Appendix B.4 for more details on this computation. We now present the theoretical results on the particle gradient descent, which corresponds to the blue dashed lines in Figure 6. The reader is invited to refer to this figure any time he needs to get a hold on the broader picture.

#### 3.3.1. Theoretical Results

The main idea of these papers [3,23] boils down to the following observation: the minimisation of function (7) is a peculiar case of a more general problem, formulated in terms of measure of the lifted space Ω. The space is more precisely $P_{2}\left(\Omega \right)$ subset of $M\left(\Omega \right)$, namely the space of probabilities with finite second moments endowed with the 2-Wasserstein metric i.e., the optimal transport distance: see Appendix B.5 for more details. Hence, the lift of the unknown $m\in M^{+}\left(X\right)$ to $\mu \in P_{2}\left(\Omega \right)$ enables the removal of the asymmetry for discrete measures between position x∈X and amplitude $a\in R^{+}$ by lifting $a\delta_{x}$ to $\delta_{\left(a,x\right)}$. The lifted functional now writes down for parameter λ>0:

(9) $$\begin{array}{c} \forall \mu \in P_{2}\left(\Omega \right),\;F\left(\mu \right)\overset{\mathrm{def}.}{=}{\left\|y-\tilde{\Phi }\mu \right\|}_{H}^{2}+\lambda \tilde{V}\left(\mu \right) \end{array}$$

where $\tilde{\Phi }\mu \overset{\mathrm{def}.}{=}\int_{\Omega }\phi \left(a,x\right)\mu \left(a,x\right)$ for $\phi \left(a,x\right)\overset{\mathrm{def}.}{=}a\varphi \left(x\right)$ and $\tilde{V}$ is the TV-norm on the spatial component of the measure μ. The functional is non-convex, its Fréchet differential is denoted $F^{\prime }$, and for u∈Ω:

$$\begin{array}{c} F^{\prime }\left(\mu \right)\left(u\right)\overset{\mathrm{def}.}{=}{\langle {\tilde{R}}^{\prime }\left(\mu \right),\phi \left(u\right)\rangle }_{H}+\lambda \end{array}$$

with ${\tilde{R}}^{\prime }\overset{\mathrm{def}.}{=}{\left\|y-\int_{\Omega }\nabla \phi \left(a,x\right)\mu \left(a,x\right)\right\|}_{H}^{2}$. Then, a discrete measure $\mu_{N}\overset{\mathrm{def}.}{=}\frac{1}{N}\sum_{i}^{N}\delta_{a_{i},x_{i}}$ of $P_{2}\left(\Omega \right)$ can also be seen as an element of $\Omega^{N}$ from the standpoint of its components $\left(a_{i},x_{i}\right)$. It allows the authors of [3,23] to perform a precise characterisation of the source recovery conditions, through the measures and the tools of optimal transport such as gradient flow (see below).

Then, one may run a gradient descent on the amplitudes and positions $\left(a_{i},x_{i}\right)\in {\left(R^{+}\times X\right)}^{N}$ of the measure $\mu_{N}$, in order to exploit the differentiability of the kernel φ. Note that the measure $\mu_{N}$ is over-parametrized, i.e., its number of δ-peaks is larger compared to the number of spikes of the ground-truth measure: thus, the particles, namely the δ-peaks of the space Ω, are covering the domain X for their spatial part.as an example, where $\mu_{N}$ is plotted in red dots.

Before giving the main results, we need to clarify the generalised notion of gradient descent to measure function called the *gradient flow* [35,36] from optimal transport theory, the main ingredient in the particle gradient descent. Letting $F:R^{d}\to R$ be the objective function with certain regularity, a gradient flow describes the evolution of a curve x(t) such that its starting point at t=0 is $x_{0}\in R^{d}$, evolving by choosing at any time *t* in the direction that decreases the function *F* the most [36]:

$$\begin{array}{c} \left\{\begin{array}{c} x^{\prime }\left(t\right)=-\nabla F\left(x\left(t\right)\right)\;\mathrm{pour}\;t>0\\ x\left(0\right)=x_{0}. \end{array}\right. \end{array}$$

The interest of gradient flow is its extension to spaces *X* with no differentiable structure. In the differentiable case, one can consider the discretisation of the gradient flow i.e., the sequence defined for a step-size τ>0, $k\in N^{*}$:

$$\begin{array}{c} x_{k+1}^{\tau }\in \underset{x\in X}{\mathrm{argmin}}F\left(x\right)+\frac{|x-x_{k}^{\tau }{|}^{2}}{2\tau }. \end{array}$$

It is the implicit Euler scheme for the equation ${\left(x^{\tau }\right)}^{\prime }=-\nabla F\left(x^{\tau }\right)$, or the weaker ${\left(x^{\tau }\right)}^{\prime }\in \partial F(x^{\tau })$ if *F* is convex and non-smooth. The gradient flow is then the limit (under certain hypotheses) of the sequence ${\left(x_{k}^{\tau }\right)}_{k\geq 0}$ for τ→0 for a starting point $x_{0}\in X$. Gradient flow can be extended to metric space: indeed, for a metric space (X,d) and a map F:X→R lower semi-continuous one can define the discretisation of gradient flow by the sequence

(10) $$\begin{array}{c} x_{k+1}\in \underset{x\in \;X}{\mathrm{argmin}}F\left(x\right)+\frac{d{\left(x,x_{k}\right)}^{2}}{2\tau }. \end{array}$$

In the case of the metric space of probability measures i.e., the measures with unitary mass, the limit τ→0 of the scheme exists and converges to the unique gradient flow starting at $x_{0}$ element of the metric space. A typical case is the space of probabilities with finite second moments $P_{2}\left(\Omega \right)$, endowed with 2-Wasserstein metric, i.e., the optimal transport distance (see Appendix B.5): a gradient flow in this space $P_{2}\left(\Omega \right)$ is a curve $t\mapsto \mu_{t}$ called a *Wasserstein gradient flow* starting at $\mu_{0}\in P_{2}\left(\Omega \right)$, for all $t\in R^{+}$, one has $\mu_{t}\in P_{2}\left(\Omega \right)$, obeying the partial differential equation in the sense of distributions:

(11) $$\begin{array}{c} \partial_{t}\mu_{t}=-\mathrm{div}\left(\mu_{t}\nabla F^{\prime }\left(\mu_{t}\right)\right). \end{array}$$

Recall that $\mathrm{div}\left(m\right)=\sum_{i=1}^{d}\frac{\partial m}{\partial x_{d}}$ for all $m\in M\left(X\right)$, derivatives ought to be understood in the distributional sense. This equation ensures the conservation of the mass, namely, at each time t>0, one has $|\mu_{t}\left|\left(\Omega \right)=\right|\mu_{0}|\left(\Omega \right)$. Hence, despite the lack of differentiability structure of $P_{2}\left(\Omega \right)$ which forbids straightforward application of a classical gradient-based algorithm, one can perform an optimisation on the space through gradient flow to reach a *minimum* of *F* by discretizing (11).

The interesting case of a gradient flow in $P_{2}\left(\Omega \right)$ is the flow starting at $\mu_{N,0}\overset{\mathrm{def}.}{=}1/N\sum_{i=1}^{N}\delta_{\left(a_{i}^{0},x_{i}^{0}\right)}$, uniquely defined by Equation (11), which writes down for all $t\in R^{+}$: $\mu_{N,t}\overset{\mathrm{def}.}{=}1/N\sum_{i=1}^{N}\delta_{\left(a_{i}\left(t\right),x_{i}\left(t\right)\right)}$, where $a_{i}:R^{+}\to R^{+}$ and $x_{i}:R^{+}\to X$ are continuous maps. This path ${\left(\mu_{N,t}\right)}_{t\geq 0}$ is a Wasserstein gradient flow, and uses *N* Dirac measures over Ω to optimise the objective function *F* in (9). When the number of particles *N* goes to infinity and if $\mu_{N,0}$ converges to some $\mu_{0}\in P_{2}\left(X\right)$, the gradient flow ${\left(\mu_{N,t}\right)}_{t\geq 0}$ converges to the unique Wasserstein gradient flow of *F* starting from $\mu_{0}$, described by the time-dependent density ${\left(\mu_{t}\right)}_{t\geq 0}$ valued in $P_{2}\left(X\right)$ obeying the latter partial differential Equation (11).

For these non-convex gradient flows, the authors of [3] give a consistent result for gradient based optimisation methods: under a certain hypothesis, the gradient flow ${\left(\mu_{N,t}\right)}_{t\geq 0}$ converges to global *minima* in the over-parametrization limit i.e., for N→+∞. It relies on two important assumptions that prevent the optimisation from being blocked in non-optimal points:

- homogeneity (A function *f* between vector spaces is positively *p*-homogeneous if, for λ>0 and argument *x*, one has $f\left(\lambda x\right)=\lambda^{d}f\left(x\right)$.) of ϕ in order to select the correct magnitude for each feature, or at least partially 1-homogeneity (i.e., boundedness of φ in [3]);
- diversity in the initialisation of parameters, in order to explore all combinations of features. Too few or too close particles will not reach all source peaks and will only yield local *minima*.

We can then introduce the fundamental result for the many particle limits [3], the mean-field limits of gradient flows ${\left(\mu_{N,t}\right)}_{t\geq 0}$, despite the lack of convexity of these gradient flows:

**Theorem** **2** **(Global convergence—informal).**If the initialisation $\mu_{N,0}$ is such that $\mu_{0}\overset{\mathrm{def}.}{=}\lim_{N\to +\infty }\mu_{N,0}$ support separates (The support of a measure m is the complement of the largest open set on which m vanishes. In an ambient space X, we say that a set C separates the sets A and B if any continuous path in X with endpoints in A and B intersects C.) {−∞}×X from {+∞}×X then the gradient flow $\mu_{t}$ weakly-* (see Appendix B.1) converges in $P_{2}\left(\Omega \right)$ to a global minimum *of F, and we also have:*

$$\begin{array}{cc} \lim_{N,t\to \infty }F\left(\mu_{N,t}\right) & =\min_{m\in M^{+}\left(X\right)}J\left(m\right). \end{array}$$

Limits can be interchanged; the interested reader might take a look at [3] for precise statements and exact hypothesis (boundary conditions, ‘Sard-type’ regularity e.g., φ is *d*-times continuously differentiable, etc).

Since we have a convergence result, we can then investigate the numerical implementation. This optimisation problem is tractable thanks to the Conic Particle Gradient Descent algorithm [23] denoted CPGD: the proposed framework involves a slightly different gradient flow ${\left(\nu_{t}\right)}_{t\geq 0}$ defined through a projection of ${\left(\mu_{t}\right)}_{t\geq 0}$ onto $M^{+}\left(X\right)$. This new gradient flow ${\left(\nu_{t}\right)}_{t\geq 0}$ is defined for a specific metric in $M^{+}\left(X\right)$, which is now a trade-off between Wasserstein and Fisher–Rao (also called Hellinger metric.) metric [23], it is then called a *Wasserstein–Fisher–Rao gradient flow*. Then, the Wasserstein–Fisher–Rao gradient flow starting at $\nu_{N,0}\overset{\mathrm{def}.}{=}\sum_{i=1}^{N}a_{i}^{0}\delta_{x_{i}^{0}}$ in $M^{+}\left(X\right)$ writes down $t\mapsto \nu_{N,t}\overset{\mathrm{def}.}{=}\frac{1}{N}\sum_{i=1}^{N}{r_{i}\left(t\right)}^{2}\delta_{x_{i}\left(t\right)}$ in $M^{+}\left(X\right)$, rather than the Wasserstein flow $t\mapsto \mu_{N,t}\overset{\mathrm{def}.}{=}\frac{1}{N}\sum_{i=1}^{N}\delta_{r_{i}\left(t\right),x_{i}\left(t\right)}$ starting at $\mu_{N,0}$ in $P_{2}\left(\Omega \right)$. The partial differential equation of a Wasserstein–Fisher–Rao flow writes down:

(12) $$\begin{array}{c} \partial_{t}\nu_{t}=-4\alpha \nu_{t}T_{\lambda }\left(\nu_{t}\right)+\beta \mathrm{div}\left(\nu_{t}\nabla J_{\nu }^{\prime }\left(\nu_{t}\right)\right) \end{array}$$

for the two parameters α,β>0 arising from the cone metric, α tunes the Fisher–Rao metric weight, while β tunes the Wasserstein metric one. All statements on convergence could be made alternatively on $\mu_{t}$ or $\nu_{t}$, and we have indeed the same theorem:

**Theorem** **3** **(Global convergence—informal).***If $\nu_{0}$ has full support (its support is the whole set X) and ${\left(\nu_{t}\right)}_{t\geq 0}$ converges for t→+∞, then the limit is a global* minimum of J. If $\nu_{N,0}\underset{N\to +\infty }{\to }\nu_{0}$ in the weak-* sense, then:

$$\begin{array}{cc} \lim_{N,t\to \infty }J\left(\nu_{N,t}\right) & =\min_{m\in M^{+}\left(X\right)}J\left(m\right). \end{array}$$

**Summary (3rd algorithm theoretical aspects):** we introduced the proposed solution of [3,23], namely approximating the source measure by a discrete non-convex objective function of amplitudes and positions. The analytical study of the discrete function is an uphill problem and could be tackled thanks to the recast of the problem in the space of measures. Then, we exhibited the theoretical framework on gradient flows, understood in the sense of generalisation of gradient descent in the space of measures. Eventually, we presented the convergence results of the gradient flow denoted ${\left(\nu_{t}\right)}_{t}$ towards the *minimum* of the BLASSO, thus enabling results for the convergence. Gradient descent on the discrete objective approximates well the gradient flow dynamic and can then benefit from the convergence results exhibited before.

We now discuss the numerical results of the particle gradient descent. The reader is advised to take a look at Figure 6, more precisely at red and green ellipses, to get a grasp on the numerical part.

#### 3.3.2. Numerical Results

We recall that a gradient flow ${\left(\nu_{N,t}\right)}_{t\geq 0}$ starting at $\overset{\mathrm{def}.}{=}1/N\sum_{i=1}^{N}{\left(r_{i}^{\left(0\right)}\right)}^{2}\delta_{x_{i}^{\left(0\right)}}$ can be seen as a (time continuous) generalisation of gradient descent in the space of measures, allowing precise theoretical statements on the recovery conditions. To approach this gradient flow, we use the Conic Particle Gradient Descent algorithm [23] denoted CPGD: the point is to discretise the evolution of the gradient flow $t\mapsto \nu_{N,t}$ through a numerical scheme on (12). This consists of a gradient descent on the amplitudes *r* and positions *x* through the gradient of the functional $F_{N}$ in Equation (8), a strategy which approximates well the dynamic of the gradient flow [23].

This choice of gradient with the cone metric enables multiplicative updates in *r* and additive in *x*, the two updates being independent of each other. Then, the algorithm consists of a gradient descent with the definition of $r_{i}^{\prime }\left(t\right)$ and $x_{i}^{\prime }\left(t\right)$ according to [2,23]:

(13) $$\begin{array}{c} \left\{\begin{array}{c} r_{i}^{\prime }\left(t\right)=-2\alpha r_{i}\lambda \left(\eta_{\lambda }\left(x_{i}\left(t\right)\right)-1\right)\\ x_{i}^{\prime }\left(t\right)=-\beta \lambda \nabla \eta_{\lambda }\left(x_{i}\left(t\right)\right) \end{array}\right. \end{array}$$

thanks to a gradient in Equation (8), for the mirror retraction (The notion of *retraction* compatible with cone structure is central: in the Riemann context, a retraction is a continuous mapping that maps a tangent vector to a point on the manifold. Formally, one could see it as a way to enforce the gradient evaluation to be mapped on the manifold. See [23] for other choices of compatible retractions and more insights on these notions.) and $\eta_{\lambda }=-J_{\nu }^{\prime }/\lambda$. The structure of the CPGD is presented in Algorithm 4. Note that the multiplicative updates in *r* yields an exponential of the certificate, and that the updates of the quantities r,x are separated.

**Figure 7:**
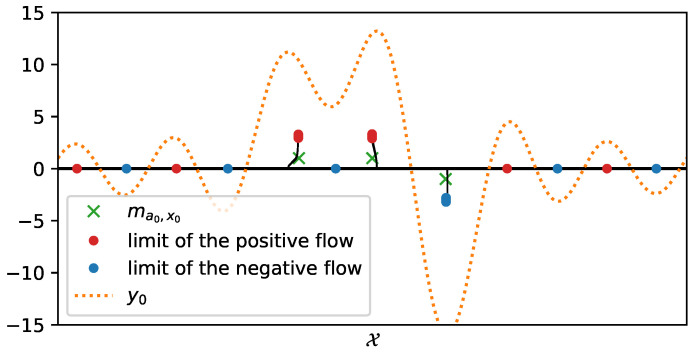
Reconstruction by *Conic Particle Gradient Descent* for a 1D Fourier operator in a noiseless setting, with the same ground-truth spikes as the former section. Implementation is an adaptation of [23], $\alpha =\beta =1\times 10^{-3}$ and λ=1 for 1000 iterations.

**Figure 8:**
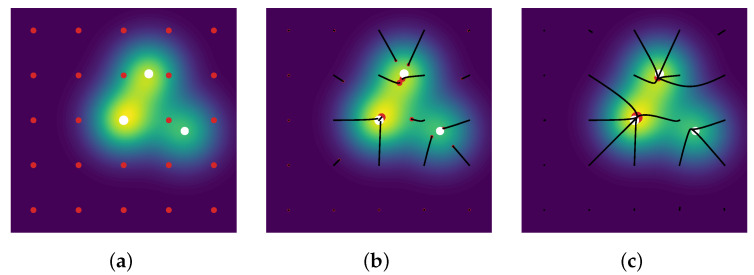
(**a**) Initialisation k=0; (**b**) mid-computation k=150; (**c**) end of the computation k=1000. *Conic Particle Gradient Descent* applied for 2D Gaussian deconvolution, the red dots are the particle measure $\nu^{\left(k\right)}$ (size of dot proportional with amplitude), the three white dots are the source measure, the image in the background is the noiseless acquisition $y_{0}$ and the black lines are the paths of the particles $\nu^{\left(k\right)}$—all the paths constitute the gradient flow ${\left(\nu_{t}\right)}_{t\geq 0}$. Implementation is an adaptation of [23], $\alpha =\beta =1\times 10^{-2}$ and λ=1.

This algorithm has rather easy and cheap iterations: to reach an accuracy of ε—i.e., a distance such as the *∞*-Wasserstein distance between the source measure $m_{a_{0},x_{0}}$ and the reconstructed measure $m^{*}$ is below ε—the CPGD yields a typical complexity cost of $\log(\varepsilon^{-1})$ rather than $\varepsilon^{-1/2}$ for convex program ([23] Theorem 4.2). A reconstruction from the latter 1D Fourier measurements is plotted in Figure 7, the reconstruction is obtained through two gradient flows, the former on the positive measures to recover the positive δ-peaks of the ground-truth and the latter on the negative measures to recover the negative one: the merging of the two results gives the reconstructed δ-peaks. The noiseless reconstruction (See our GitHub repository for our implementation: https://github.com/XeBasTeX, accessed on 30 November 2021) for 2D Gaussian convolution with the same setting as the Frank–Wolfe section is plotted in Figure 8. One can see that the spikes are well-recovered as some non-zero red and blue particles cluster around the three δ-peaks.
**Algorithm 4.** Conic particle gradient descent algorithm.
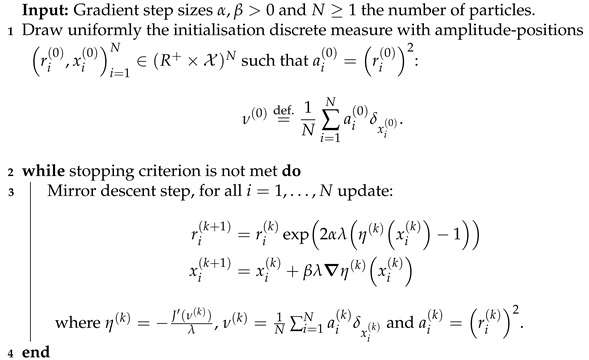


**Summary (3rd algorithm numerical aspects):** the gradient flow ${\left(\nu_{t}\right)}_{t}$ is computable by the Conic Particle Gradient Descent algorithm, consisting in an estimation through a gradient (w.r.t. cone metric) descent on both amplitudes and positions of an over-parametrised measure, namely a measure with a fixed number of δ-peaks exceeding the source’s one. The iterations are cheaper than the SFW presented before, but the CPGD lacks guarantees in a low-noise regime.

To sum up all the pros and cons of these algorithms, we give Table 1 for a quick digest. Since the CPGD lacks guarantees on the global optimality of its output, the following section will use the conditional gradient and more precisely the *Sliding Frank-Wolfe* in order to tackle the SMLM super-resolution problem.

## 4. Applications and Results in SMLM Imaging

As an illustration of off-the-grid applications’ in this review, we propose to solve the super-resolution problem, aiming to retrieve biological structures at very small scales.

### 4.1. Metrics of Quality of Reconstruction

If one has access to the ground-truth i.e., the real position of the point sources, one is able to assess the quality of the reconstruction by:

- detection metrics, such as the Jaccard index;
- quality of reconstruction metrics, such as the $L^{2}$ norm in the discrete case.

Detection metrics can be applied to the off-the-grid output in a straightforward manner. We will rather focus in this part on the ‘quality of reconstruction’ metric. Any of the former algorithms returns a list of Dirac measures, which can be compared with the ground-truth measure $m_{a_{0},x_{0}}$. This comparison cannot be done with discrete tools, such as the $L^{2}$ norm of the reconstructed acquisitions: we cannot compare an element of $M\left(X\right)$ with $L^{2}\left(X\right)$. Examining the $L^{2}$ norm of the $x_{i}$ vector of reconstructed positions against the $x_{0,i}$ vector is not sufficient either: we need the same number of elements for *x* and $x_{0}$, we have to sort the vector of positions, and we have no guarantee that the matching of one position of *x* with another of $x_{0}$ is the right one.

Hence, a distance on the measure space is the good tool of comparison. We will use in the following the Wasserstein 1-distance $W_{1}$ [37]: see Appendix B.5 for some recall on the useful definition and more insights on the optimal transport setting used in this section. The Wasserstein distance with measures of equal mass is defined (Actually, it is well-defined on the subset $X\overset{\mathrm{def}.}{=}\left\{m\in M\left(X\right),\left|m\right|\left(X\right)\leq y_{H}^{2}/2\lambda \right\}$ because only the bounded subset of $M\left(X\right)$ are metrizable for the weak-* topology, so we have to restrain the set of measures to *X* in order to reach a Polish space i.e., the convenient framework for this OT-based metric, see Appendix B.5. Since all solutions of the BLASSO belong to *X* [8], we will keep this slight abuse of notation in the rest of the paper.) as:

**Definition** **7** **(Balanced optimal transport).** *For 0≤p<+∞ and $m_{1},m_{2}\in M\left(X\right)$ such that $|m_{1}\left|\left(X\right)=\right|m_{2}|\left(X\right)$, the p-Wasserstein distance is written:*

(14) $$\begin{array}{c} W_{p}\left(m_{1},m_{2}\right)\overset{\mathrm{def}.}{=}{\left(\min_{\gamma \in \Gamma (m_{1},m_{2})}\int_{X\times X}{\left|u-v\right|}^{p}\;d\gamma \left(u,v\right)\right)}^{1/p}. \end{array}$$

$\Gamma (m_{1},m_{2})$ is the set of transport plans between $m_{1}$ and $m_{2}$, one can take a look at Appendix B.5 for more insights on this notion. However, this notion is not sufficient for our application since the metric can only take measures of equal masses (i.e., equal TV-norm) as an input. In the case of a discrete measure, we recall that mass is simply the sum of the modulus of individual amplitudes: hence, in general, we cannot compare a source measure and a reconstructed measure with differing amplitudes. The classic solution is then to distribute the unit mass, divided by the number of spikes, uniformly over each δ-peak of the discrete measure. Still, it would be way more convenient to incorporate the case of differing masses in the metric. The proper metric to compare two measures of different masses is called the Kantorovtich–Rubinstein norm also referred as the Flat Metric [37,38,39].

**Definition** **8** **(Unbalanced optimal transport).** *Let us denote $m\in M\left(X\right)$ of finite first moment and τ>0, and the following quantity is called Kantorovtich–Rubinstein norm:*

$$\begin{array}{c} F_{\tau }\left(m\right)\overset{\mathrm{def}.}{=}\underset{f\in C_{b}\left(X\right)}{\sup}\left(\int_{X}f\;dm,{\left\|f\right\|}_{\infty ,X}\leq \tau ,f\;\mathrm{Lipschitz},{\left\|f\right\|}_{\mathrm{Lip}}\leq 1\right) \end{array}$$

*where ${\left\|f\right\|}_{\mathrm{Lip}}$ is the Lipschitz constant of f. We then define the* Flat Metric *$d_{\tau }$ for $m_{1},m_{2}\in M\left(X\right)$ of finite first moments:*

$$\begin{array}{c} d_{\tau }\left(m_{1},m_{2}\right)\overset{\mathrm{def}.}{=}F_{\tau }\left(m_{1}-m_{2}\right). \end{array}$$

The parameter τ is homogeneous to a distance, and it is understood in the optimal transport sense as the cost of creating/destroying a Dirac measure. The Flat Metric coincides with the 1-Wasserstein distance, for $m_{1}$, $m_{2}$ of equal masses, when τ→+∞ [38]; it also coincides with the total variation norm of $m_{1}-m_{2}$ when τ→0. Then, it may be seen as an interpolation between the total variation norm and the 1-Wasserstein norm. Moreover, when the number of δ-peaks is correctly estimated, the Flat Metric stands for the mean error in terms of localisation and is similar to the RMSE [39]. Eventually, the Flat metric can be extended to discrete reconstruction i.e., images on a fine grid; this metric is then a method applicable to discrete reconstruction, namely images with a finer grid.

To sum up, there are two possibilities if one wants to compare the reconstructed measure and the ground-truth one:

- let the source measure be composed of *N* spikes, we set the amplitude of each δ-peak at 1/N. We apply the same procedure to the reconstructed (with differing or not number of spikes), hence dividing uniformly the unit mass over all the δ-peaks of the considered measure. Therefore, the reconstructed luminosity is not considered as relevant and discarded: we can compute directly the 1-Wasserstein distance, since it is equal to the Flat Metric in this case;
- we want to account for the luminosity, and we use the Flat Metric to compare the reconstructed measure against the ground-truth one.

**Summary:** classic quality of reconstruction metrics such as the $L^{2}\left(X\right)$ norm cannot be straightforwardly applied to off-the-grid reconstruction. Instead, one could use optimal transport score such as the Flat Metric: it accounts for both amplitude and position reconstructions, while it can be easily extended to discrete reconstruction (images on a fine grid).

### 4.2. Results for an SMLM Stack

In super-resolution for biomedical imaging, one wants to retrieve some fine scale details to better study biological structures of interest. Indeed, the studied bodies are generally smaller than the Rayleigh limit at 200 nm, a length at which the phenomenon of light diffraction comes into play. This diffraction causes a blurring of the image, which can be described as a convolution of the image by the PSF mentioned above. Hence, we want to perform a *deconvolution* i.e., remove the blur of diffraction to get a super-resolved image. It is worth noticing that other imaging systems exist, for which the inverse problems to solve are a bit different from deconvolution: e.g., Nuclear Magnetic Resonance spectroscopy with Fourier measurements [40], MA-TIRF with Laplace [13].

In order to enhance spatial resolution over standard diffraction-limited microscopy techniques and allow imaging of biological structures below the Rayleigh criterion, one can use SMLM, which stands for ‘Single Molecule Localisation Microscopy’. It is a compelling technique in fluorescence microscopy to tackle the super-resolution problem [41]. It requires photoactivable fluorophores with, roughly speaking two states, for example ‘*On*’ and ‘*Off*’. These molecules are therefore only visible on the acquisitions in the ‘*On*’ case, and the idea is then to light up some molecules in the sample to make the acquisition and to be able to locate them precisely; the fluorescent molecules are bound to the biological structure and, since only a few molecules are emitting in one frame, the resulting image is rather sparse, which allows accurate localisation. This process is repeated until all the molecules have been lit and imaged. All the positions of the imaged molecules frame-by-frame can then be put together to form a super-resolved image that go below the diffraction barrier, ridden of the degradation by the process of acquisition (blur, noise, etc.). The quality of the image reconstruction is naturally limited by the number of acquisitions necessary to reconstruct the image, which implies a cost in time (precious insofar as the organism studied moves) and in physical memory and by the density of fluorophores lit at each stage. Indeed, there is a risk of overlap hindering the localisation of the molecules since the separation criterion is not matched.

Off-the-grid methods can be applied to any SMLM stack with only the knowledge of the forward operator, the acquisition system’s PSF in this case. In this review, a gridless method based on Sliding Frank–Wolfe is tested on an 2D SMLM acquisition stack from the 2013 EPFL Challenge (https://srm.epfl.ch/DatasetPage?name=MT0.N1.HD, accessed on 30 November 2021). For this purpose, we consider the first image of the stack, locate the source points, and store the coordinates of these points. Then, we move on to the second image, we locate the source points, and so on. Note that off-the-grid method with this variational approach is not the only method taking advantage of a continuous domain like the PSF-fitting such as DAOSTORM [42], etc.

Deconvolution is a first challenge to solve this inverse problem, but we must also take into account the noise. One has to deal with three main types of noise on these acquisitions:

- photon noise (also known as shot noise or quantum noise) is due to the quantum nature of light. It arises from the fact that fluorophores emit photons randomly, so that, between *t* and t+1 (exposure time), a variable number of photons have been emitted, and therefore a variable number of photons have been collected by the sensor. Thus, the amplitude of the electrical signal generated in the sensor (at each pixel) fluctuates according to a Poisson statistic;
- the dark current is a phenomenon due to the natural agitation of electrons. This natural agitation is sufficient to occasionally eject an electron from the valence band to the conduction band without any photoelectric effect. Additional charges are therefore created which interfere with the signal. The number of electrons generated by thermal agitation follows a Poisson distribution;
- amplification and readout noise. This noise is produced by the electronic circuit that amplifies and converts the electron packets into voltage. It is generally modelled by a Gaussian noise.

Thus, we have several noises that pollute each of the observed images. To deal with this ill-posed inverse problem, we use the results on BLASSO, with the least-squares term as the data-fitting term and the TV norm as the regulariser of the inverse problem. In the Bayesian approach, the least-square term is modelling the maximum of likelihood when the acquisition is polluted by Gaussian noise, hence our model is making the approximation of Gaussian noise. Measurements are discrete so at each image one has to deal with images with $N_{1}\times N_{2}$ pixels, each of them with size $\left(b_{1},b_{2}\right)$. Let $\left(c_{i,1},c_{i,2}\right)$ be the centre of the *i*th pixel, and we denote the *i*th camera pixels by

$$\begin{array}{c} \Omega_{i}\overset{\mathrm{def}.}{=}\left(c_{i,1},c_{i,2}\right)+\left[-\frac{b_{1}}{2N_{1}},\frac{b_{1}}{2N_{1}}\right]\times \left[-\frac{b_{2}}{2N_{2}},\frac{b_{2}}{2N_{2}}\right]. \end{array}$$

We can then clarify the forward operator $\Phi :m\mapsto R^{N_{1}N_{2}}$ which encapsulates the integration over camera pixels [13]; indeed, with the evaluation of the discrete Gaussian kernel φ with standard deviations σ, for $i\in \{1,\cdots ,N_{1}N_{2}\}$:

$$\begin{array}{c} {\left[\varphi \left(x\right)\right]}_{i}\overset{\mathrm{def}.}{=}\frac{1}{2\pi \sigma^{2}}\int_{\Omega_{i}}e^{-\left(\frac{{\left(x_{1}-s_{1}\right)}^{2}}{2\sigma^{2}}+\frac{{\left(x_{2}-s_{2}\right)}^{2}}{2\sigma^{2}}\right)}\;ds_{1}s_{2}. \end{array}$$

**Figure 9:**
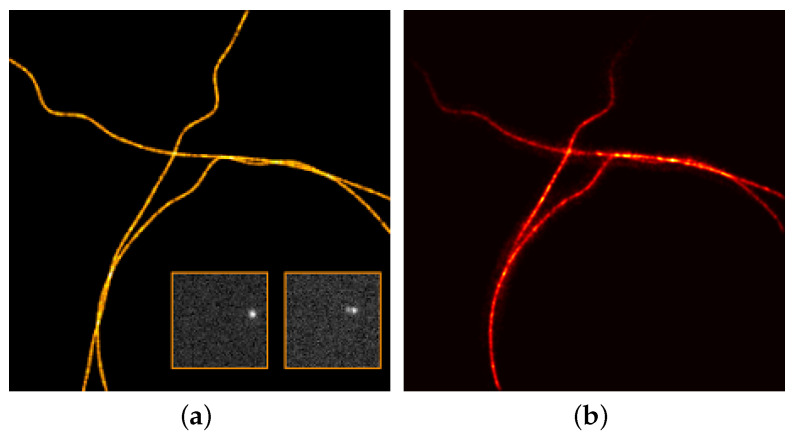
(**a**) Ground-truth tubulins, two excerpts of the stack in the square below: convolution + all noise described before; (**b**) reconstructed measure by *Sliding Frank-Wolfe* visualised through Gaussian kernel with a smaller σ (see text).

In the SMLM data set, one has the PSF standard deviation σ=149.39nm and $N_{1}=N_{2}=100\;\mathrm{nm}$. The reconstruction is performed by our implementation of the Sliding Frank–Wolfe in python (See our GitHub repository for our PyTorch implementation: https://github.com/XeBasTeX, accessed on 30 November 2021) insofar as it is the more robust method available: indeed, it works with Gaussian kernel, and it has proven results in a noise regime, etc. The results are presented in Figure 9. The stack of 2500 images of 64×64 is qualified as high density with high SNR: the number of activated fluorophores is quite important, and the noise is not negligible (see the EPFL Challenge page for more insights.).

A flat metric between the reconstructed measure $m_{a,x}$ and the ground-truth measure $m_{a_{0},x_{0}}$ is then computed, and it reaches $d_{\tau }\left(m_{a,x},m_{a_{0},x_{0}}\right)=1.7\times 10^{-2}$. The reconstruction is convincing and well captures the fine details of the biological structures, and one can clearly see the interweaving tubulins in the right part of the image.

Note that an interesting feature of the gridless reconstruction is that, once the Radon measure is computed, it is straightforward to plot it through any operator on a fine grid of one choice. Indeed, as one cannot represent a discrete measure *m*, we rather plot Φm, where Φ is the PSF with a slightly smaller variance, in order to clearly see the deconvolution. In all of our reconstructions, we convolve the reconstruction through the PSF with variance σ/6 and plot it on a grid 32 times finer. As a matter of comparison, discrete methods are performed for a fixed fine grid, and, if one wants a finer reconstruction, one has to recompute everything.

**Figure 10:**
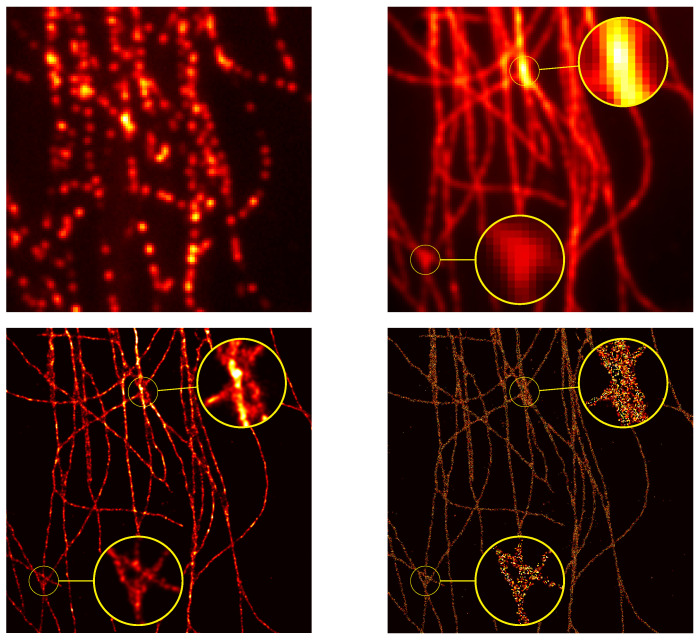
(**top left**) Excerpt of the stack; (**top right**) mean of the stack; (**bottom left**) reconstruction by off-the-grid method; (**bottom right**) Deep-STORM.

We finally test the off-the-grid reconstruction on a real data set of tubulins with high density molecules, provided by the 2013 IEEE ISBI SMLM challenge. In this stack of 500 frames of 128×128 pixels, the FWHM (full width at half maximum) of the acquisition system is estimated at 351.8 nm. We recall that the FWHM is the width of the Gaussian curve measured between those points on the *y*-axis, which are half the maximum amplitude, also note that it is linked to the variance σ by $\mathrm{FWHM}=2\sqrt{2\ln2}\times \sigma$. We compare the reconstruction of the off-the-grid method with the output of the Deep-STORM [43] algorithm, touted as the algorithm with the most visually compelling results. The reconstructions of the gridless method and the Deep-STORM algorithm are presented in Figure 10, where one can appreciate the reconstruction by off-the-grid on fine details. The reconstruction seems a small bit blurry compared to Deep-STORM, due to the plotting through a small spread Gaussian kernel. However, it is noteworthy that both comparisons perform well to retrieve the filaments, in particular in the enhancing yellow circles: the off-the-grid reconstruction seems to better preserve the structure compared to the Deep-STORM’s rough output. The quality of the reconstruction is notably interesting for off-the-grid reconstruction since it does not require any test sets to yield this reconstruction, contrary to Deep-STORM. The only data needed are the knowledge of (an estimation of) the forward operator, and the off-the-grid reconstruction can be then performed from any input without having to train the model on different types and levels of noise.

**Shorthand:** We tested an off-the-grid method on both SMLM synthetic and experimental data set. The gridless problem is tractable thanks to the Sliding Frank–Wolfe algorithm, and yields compelling results. The results are all the more interesting since there is only one parameter, handy to tune and robust w.r.t. noise. Thus, it can be easily adapted to any other dataset with a known acquisition operator.

## 5. Conclusions

We described in this review the off-the-grid variational settings for the sparse spike problem, through the definition of the space of signed measures $M\left(X\right)$ and the functional BLASSO defined over this set. Thanks to the trade-off between the convexity of the functional and the infinite dimensional, non-reflexive space of optimisation $M\left(X\right)$; the BLASSO can be defined to solve the sparse spike recovery problem. We review in this paper the theoretical guarantees to reach the correct *minimum* as the literature provides multiple results, in particular a sharp criterion for stable spike recovery under a low noise regime. Numerical methods to tackle the BLASSO problem were also discussed, with insights on the SDP approach, which is asymptotically exact but only suited for Fourier measurements, the Frank–Wolfe approach with known rate of convergence but a high computation load and the Conic Particle Gradient Descent with cheap iterations but lacks of guarantees. We were finally able to present the result of the off-the-grid approach with a Sliding Frank–Wolfe algorithm in the case of SMLM synthetic *data* and real data from the EPFL Challenge, and to illustrate the usefulness of these methods to recover fine-scale details.

## Figures and Tables

**Table 1:** Pros and cons for the different off-the-grid algorithm strategies, Semi-definite programming (SDP) vs. *Sliding Frank-Wolfe* (SFW) algorithm vs. *Conic Particle Gradient Descent* (CPGD).

Algorithm	Operator	Space X	Convergence Rate	Computation Time	Tuning Parameters
SDP [10]	Fourier	Torus $T^{d}$	Asymptotic	Mild	λ
SFW [11,13]	All	Any compact	Sublinear	Long	λ
CPGD [23]	All	Torus $T^{d}$	log(ε)	Quick	λ,α,β

## Data Availability

All the codes are available at the git repository https://github.com/XeBasTeX/Journal-of-Imaging-2021 or its mirror https://gitlab.inria.fr/blaville/Journal-of-Imaging-2021 (accessed on 30 November 2021).

## Abbreviations

The following abbreviations are used in this manuscript:

PSF	Point Spread Function
FWHM	Full Width at Half Maximum
SNR	Signal-to-Noise Ratio
LASSO	Least Absolute Shrinkage and Selection Operator
BLASSO	Beurling-LASSO
SFW	Sliding Frank–Wolfe
OT	Optimal Transport
CPGD	Conic Particle Gradient Descent
SMLM	Single Molecule Localisation Microscopy

## Appendix A. Notations Table

**Table A1:** Main notations used in the review.

*d*	dimension of the ambient space
X	ambient space of the spike positions e.g., the torus, ${\left[0,1\right]}^{d}$, $R^{d}$, etc.
$C_{0}\left(X\right)$	space of evanescent continuous functions
$M\left(X\right)$	space of signed Radon measures
$M^{+}\left(X\right)$	space of non-negative Radon measures
$M_{C}\left(X\right)$	space of complex Radon measures
H	Hilbert space, typically $L^{2}\left(X\right)$
*m*	Radon measure
|m|(X)	TV norm of *m*
δ	Dirac measure
a,x	respectively amplitudes and positions of the spike $a\delta_{x}$
*N*	number of Dirac measures in a discrete measure
λ	regularisation parameter in (𝒫_λ_(*y*))
Φ	forward acquisition operator with kernel φ and adjoint $\Phi^{*}$
$F_{n}$	forward Fourier acquisition operator with *n* measurements
$p_{\lambda }$	solution of the dual problem (𝒟_λ_(*y*))
$\eta_{\lambda }$	dual certificate of (𝒫_λ_(*y*))
$T_{\lambda }$	BLASSO (𝒫_λ_(*y*)) functional
Ω	Lifted space $\Omega \overset{\mathrm{def}.}{=}R^{+}\times X$
*J*	BLASSO functional on $M^{+}\left(X\right)$
*R*	data-term $R:M\left(X\right)\to H$
$F_{N}$	‘discrete’ functional on $\Omega^{N}$
*F*	functional on $M\left(\Omega \right)$
${\left(\mu_{t}\right)}_{t}$, ${\left(\nu_{t}\right)}_{t}$	Gradient flows respectively in $P_{2}\left(\Omega \right)$ and $M^{+}\left(X\right)$
α, β	Cone metric/ Fisher–Rao–Wasserstein tuning parameters
$W_{p}$	Wasserstein distance of order *p*
$P_{2}\left(\Omega \right)$	space of probability measures with 2nd moment endowed with $W_{2}$

## Appendix B. Useful Definitions and Notions

### Appendix B.1. Details on Functional Analysis

**Definition** **A1.**
*Two Radon measures μ and ν of $M\left(X\right)$ are called singular if there exists two disjoint subsets A, B of the σ-algebra of X whose union is X, such that μ is zero on all measurable subsets of B while ν is zero on all measurable subsets of A.*

**Proposition** **A1**
**(Jordan decomposition).** *The Jordan decomposition states for every measure $\mu \in M\left(X\right)$ the existence of two non-negative Radon measures $\mu^{+}$, $\mu^{-}\in M^{+}\left(X\right)$ which are singular and such that $\mu =\mu^{+}-\mu^{-}$.*

**Definition** **A2**
**(weak-* topology).** *Loosely speaking, weak-* convergence is convergence locally on average. We say that a sequence of Radon measures ${\left(m_{n}\right)}_{n\geq 0}$ weakly-* converges towards $m\in M\left(X\right)$ if and only if for all $f\in C_{0}\left(X\right)$:*

$$\begin{array}{c} \int_{X}f\;dm_{n}\underset{n\to +\infty }{\to }\int_{X}f\;dm. \end{array}$$

*We note $m_{n}\overset{*}{⇀}m$, and it is also called the vague convergence.*

**Definition** **A3.**
*A vector space E is said to be reflexive if the bi-dual $E^{**}$ is identified with E.*

**Remark** **A1.**
*Since $C_{0}\left(X\right)$ is not a reflexive space for its norm supremum, the dual of $M\left(X\right)$ for the topology induced by its TV norm is a complicated space, strictly larger [44] than $C_{0}\left(X\right)$. However, if $M\left(X\right)$ is endowed with the weak-* topology, then $M\left(X\right)$ is a locally convex space whose dual is $C_{0}\left(X\right)$ [8].*

We also precise the notion of metrisability for the sake of the optimal transport part:

**Definition** **A4.**
*A topological space (E,T) is said to be metrisable if there exists a distance d:E×E→[0,+∞[ such that the topology induced by d is T.*

$\left(M\left(X\right),*\right)$ is not a first-countable space, then it is not a metrisable space. To get a hold on that:

**Lemma** **A1.**
*If E is a Banach, the weak-* topology is not metrisable on $E^{*}$, except if E is of finite dimension.*

Nonetheless, all bounded subsets of $M\left(X\right)$ are metrisable for the weak-* topology. This property is of upmost importance for the definitions of classic OT metrics such as the Wasserstein distance and for the proof of Γ-convergence of the LASSO to the BLASSO [7,37]. To sum-up all the properties of the different topologies, we give the following Table A2:

**Table A2:** Algebraic properties of $M\left(X\right)$ for its two main topologies.

Properties	TV Topology	Weak-* Topology
Completeness	Yes	On its bounded subset
Separability	No	Yes
Reflexivity	No	Yes
Metrisable	Yes	On its bounded subset
Polish space (see Appendix B.5)	No	On its bounded subset

### Appendix B.2. Proof of the Fenchel Dual

**Proposition** **A2.**
*Let be the problem:*

$$\begin{array}{c} \underset{{\left\|\phi^{*}p\right\|}_{\infty ,X}\leq 1}{\mathrm{argmax}}{\langle y,p\rangle }_{H}-\frac{\lambda }{2}{\left\|p\right\|}_{H}^{2}. \end{array}$$

*It is the dual problem of the BLASSO (𝒫_λ_(*y*)).*

**Proof.** We will apply results from [20] (Remark 4.2), with a little caveat: the Banach space *V* should be reflexive, which is clearly not the case here with $V=M\left(X\right)$. However, the reflexive hypothesis is only needed for the sake of existence proof. Since we already proved the solution’s existence, this reflexivity hypothesis is not needed in our case. Back to the Remark 4.2, it states, for Λ:V→Y linear, F:V→R and V:Y→R convex that the primal problem:

$$\begin{array}{c} \underset{u\in V}{\inf}F\left(u\right)+G\left(\Lambda u\right) \end{array}$$

has a dual problem which writes down:

(A1) $$\begin{array}{c} \underset{p^{*}\in Y^{*}}{\sup}-F^{*}\left(\Lambda^{*}p^{*}\right)-G^{*}\left(-p^{*}\right). \end{array}$$

If *u* and $p^{*}$ are respectively solutions of the primal and dual, the extremality conditions are:

$$\begin{array}{c} \left\{\begin{array}{c} \Lambda^{*}p^{*}\in \partial F\left(u\right)\\ -p^{*}\in \partial G\left(\Lambda u\right). \end{array}\right. \end{array}$$

Let use specify in our case $V\overset{\mathrm{def}.}{=}M\left(X\right)$, $Y\overset{\mathrm{def}.}{=}H$, $F\left(m\right)\overset{\mathrm{def}.}{=}\left|m\right|\left(X\right)$ and $G\left(p\right)\overset{\mathrm{def}.}{=}\frac{1}{2}{y-p}_{H}^{2}$. One can clearly see that the adjoint of *G* is $G^{*}\left(p^{*}\right)={\langle y,p^{*}\rangle }_{H}+\frac{1}{2}{p^{*}}_{H}^{2}$ for $p^{*}\in H$; in order to determine $F^{*}$ let $\psi \in V^{*}\overset{\mathrm{def}.}{=}C_{0}\left(X\right)$:

$$\begin{array}{cc} F^{*}\left(\psi \right) & =\underset{m\in M\left(X\right)}{\sup}{\langle \psi ,m\rangle }_{C_{0}\left(X\right)\times M\left(X\right)}-\left|m\right|\left(X\right)\\ \; & \geq {\langle \psi ,m\rangle }_{C_{0}\left(X\right)\times M\left(X\right)}-\left|m\right|\left(X\right),\;\forall m\in M\left(X\right). \end{array}$$

Let x∈X, and $m=\lambda \delta_{x}$ with λ>0. Then, one have:

$$\begin{array}{c} \underset{m\in M\left(X\right)}{\sup}{\langle \psi ,m\rangle }_{C_{0}\left(X\right)\times M\left(X\right)}-\left|m\right|\left(X\right)\geq \lambda \left(\psi \left(x\right)-1\right). \end{array}$$

At the limit λ→∞, one yields $F^{*}\left(\psi \right)\geq +\infty$ if ψ(x)>1. A similar result for ψ(x)<1 is obtained with the measure $m=-\lambda \delta_{x}$. One finally reaches $F^{*}\left(\psi \right)=+\infty$ if ${\left\|\psi \right\|}_{\infty ,X}>1$. Let us assume that ${\left\|\psi \right\|}_{\infty ,X}\leq 1$, first note that $F^{*}\left(\psi \right)=\sup_{m\in M\left(X\right)}{\langle \psi ,m\rangle }_{C_{0}\left(X\right)\times M\left(X\right)}-\left|m\right|\left(X\right)\geq 0$ (case m=0). Moreover,

$$\begin{array}{cc} {\langle \psi ,m\rangle }_{C_{0}\left(X\right)\times M\left(X\right)}-\left|m\right|\left(X\right) & \leq {\left\|\psi \right\|}_{\infty ,X}|m|\left(X\right)-|m|\left(X\right)\\ \; & \leq |m|\left(X\right)\left({\left\|\psi \right\|}_{\infty ,X}-1\right)\\ \; & \leq 0\;\sin\mathrm{ce}\;{\left\|\psi \right\|}_{\infty ,X}\leq 1. \end{array}$$

By introducing the sup on both sides of the last inequality, one finally get $F^{*}\left(\psi \right)=0$ if ${\left\|\psi \right\|}_{\infty ,X}\leq 1$, thus reaching the condition on the *supremum* norm. Then, from (A1), we yield the dual problem:

$$\begin{array}{c} \underset{{\left\|\Phi^{*}p^{*}\right\|}_{\infty ,X}\leq 1}{\sup}{\langle y,p^{*}\rangle }_{H}-\frac{1}{2}{{\left\|p\right\|}^{*}}_{H}^{2} \end{array}$$

and for $m^{*}$ and $p^{*}$ respectively solutions of the primal and dual, its extremality conditions are:

$$\begin{array}{c} \left\{\begin{array}{c} \Phi^{*}p^{*}\in \partial |m^{*}|\left(X\right)\\ -p^{*}=\Phi m^{*}-y. \end{array}\right. \end{array}$$

This concludes the proof. □

### Appendix B.3. Fréchet Differential of J ν ′

Let $\sigma ,\nu \in M^{+}\left(X\right)$ and ε>0. Consider the following:

$$\begin{array}{cc} J(\nu +\varepsilon \sigma ) & =R\left(\int_{X}\varphi \left(\theta \right)\left(\nu +\varepsilon \;\sigma \right)\right)+\lambda \left|\nu +\varepsilon \sigma \right|\left(X\right)\\ \; & =R\left(\int_{X}\varphi \left(\theta \right)\;d\nu \left(\theta \right)+\varepsilon \int_{X}\varphi \left(\theta \right)\;d\sigma \left(\theta \right)\right)+\lambda |\nu |\left(X\right)+\varepsilon |\sigma |\left(X\right). \end{array}$$

The TV linearity is obtained thanks to the positivity of ν, σ. Hence, the differential $J_{\nu }^{\prime }$ at point ν is given by:

$$\begin{array}{cc} {\frac{J(\nu +\varepsilon \sigma )}{d\varepsilon }|}_{\varepsilon =0} & ={\langle \int_{X}\varphi \left(\theta \right)\;d\sigma \left(\theta \right),\nabla R\left(\int_{X}\varphi \left(s\right)\;d\sigma \left(s\right)\right)\rangle }_{H}+\lambda \left|\sigma \right|\left(X\right)\\ \; & =\int_{X}{\langle \varphi \left(\theta \right),\nabla R\left(\varphi (s)\;d\sigma (s)\right)\rangle }_{H}\;d\sigma \left(\theta \right)+\lambda \left|\sigma \right|\left(X\right)\\ \; & ={\langle {\langle \varphi ,\nabla R\rangle }_{H},\sigma \rangle }_{C_{0}\left(X\right)\times M\left(X\right)}+{\langle \lambda ,\sigma \rangle }_{C_{0}\left(X\right)\times M\left(X\right)}\\ \; & ={\langle J_{\nu }^{\prime },\sigma \rangle }_{C_{0}\left(X\right)\times M\left(X\right)} \end{array}$$

### Appendix B.4. Gradient of F_N_

Let R be the data fitting term e.g., the least squares, and *h* an injective function such as the map $h:r\mapsto r^{2}$. For $r\in R_{+}^{N}$ and $\theta \in X^{N}$, we consider:

$$\begin{array}{c} F_{N}\left(r,\theta \right)=R\left(\frac{1}{N}\sum h\left(r_{i}\right)\varphi \left(\theta_{i}\right)\right)+\frac{1}{N}\sum h\left(r_{i}\right). \end{array}$$

Its differential is given by:

$$\begin{array}{cc} \;dF_{N}\left(x\right)\left(\delta x\right) & =\langle \nabla F_{N}\left(x\right),\delta x\rangle \\ \; & =\frac{1}{N}\sum \alpha {\left(r\right)}^{-1}\frac{\partial F_{N}}{\partial r_{i}}\delta r_{i}+\sum \beta {\left(r\right)}^{-1}{\langle \frac{\partial F_{N}}{\partial r_{i}},\delta \theta_{i}\rangle }_{\theta }. \end{array}$$

Moreover, we have:

$$\begin{array}{cc} \frac{\partial F_{N}}{\partial r_{i}} & =\frac{1}{N}h^{\prime }\left(r_{i}\right)\varphi \left(\theta_{i}\right)\frac{\partial R}{\partial r_{i}}\left(\frac{1}{N}\sum h\left(r_{i}\right)\varphi \left(\theta_{i}\right)\right)+\frac{\lambda }{N}h^{\prime }\left(r_{i}\right)\\ \; & =\frac{1}{N}h^{\prime }\left(r_{i}\right)\left(\varphi \left(\theta_{i}\right)\frac{\partial R}{\partial r_{i}}\left(\frac{1}{N}\sum h\left(r_{i}\right)\varphi \left(\theta_{i}\right)\right)+\lambda \right)\\ \; & =h^{\prime }\left(r_{i}\right)J_{\nu }^{\prime }\left(\theta_{i}\right). \end{array}$$

Similarly,

$$\begin{array}{cc} \frac{\partial F_{N}}{\partial r_{i}} & =\frac{1}{N}h^{\prime }\left(r_{i}\right)\varphi \left(\theta_{i}\right)\frac{\partial R}{\partial r_{i}}\left(\frac{1}{N}\sum h\left(r_{i}\right)\varphi \left(\theta_{i}\right)\right)+\frac{\lambda }{N}h^{\prime }\left(r_{i}\right)\\ \; & =h\left(r_{i}\right)\nabla J_{\nu }^{\prime }\left(\theta_{i}\right). \end{array}$$

which yields the gradient in [23]. One can simplify the final expression by introducing the certificate $\eta_{\lambda }\overset{\mathrm{def}.}{=}J_{\nu }^{\prime }/\lambda$.

### Appendix B.5. Details on Section 4

**Definition** **A5.**
*A space $\left(X,d_{p}\right)$ is a Polish space if it is separable, metrizable, and has a topology—induced by a distance—which makes the space complete.*

X is a separable Hilbert space then $\left(X,d_{p}\right)$ is a Polish metric space for $d_{p}$ a distance on $R^{d}$ restricted to X. We can also introduce:

**Definition** **A6**
**(Transport Plane).** *The non-negative measure $\gamma \in M^{+}\left(X\times X\right)$ which verifies, for all A,B∈B(X), where B(X) is the Borel σ-algebra:*

$$\begin{array}{c} \gamma \left(A\times X\right)=m_{1}\left(A\right),\;\gamma \left(B\times X\right)=m_{2}\left(B\right) \end{array}$$

*is called the transport plane between two positive measures $m_{1}$ and $m_{2}$ of same mass.*

We call $\Gamma (m_{1},m_{2})$ the set of transport planes between $m_{1}$ and $m_{2}$ [37]. Metrics of optimal transport such as the Wasserstein distance use at their core these notions, and are defined only on Polish spaces: this is why we work with the measures in *X* from [7], restriction of $M\left(X\right)$ with the weak-* topology.

**Definition** **A7**
**(Wasserstein distance).** *Let the Polish metric space $\left(X,d_{p}\right)$, and p∈[1,+∞). For any probability measures μ and ν of X, the Wasserstein distance of order p between μ and ν is defined by:*

$$\begin{array}{c} W_{p}\left(\mu ,\nu \right)={\left(\underset{\gamma \in \Gamma (\mu ,\nu )}{\inf}\int_{X}d{\left(x,y\right)}^{p}\;d\gamma \left(x,y\right)\right)}^{1/p}. \end{array}$$

We also recall the definition of moments:

**Definition** **A8.**
*If r∈N, we call moment of order r of a measure $m\in M\left(X\right)$ the quantity:*

$$\begin{array}{c} \int_{X}x^{r}\;dm\left(x\right). \end{array}$$

*We say that m is of r-finite moment if the preceding quantity is finite.*
